# Supplementary figures and images for: De novo mutational signature discovery in tumor genomes using SparseSignatures
Source: PLoS Comput Biol. 2021 Jun 28;17(6):e1009119. doi: 10.1371/journal.pcbi.1009119 (PMC8270462; doi:10.1371/journal.pcbi.1009119)

**A** Patient Counts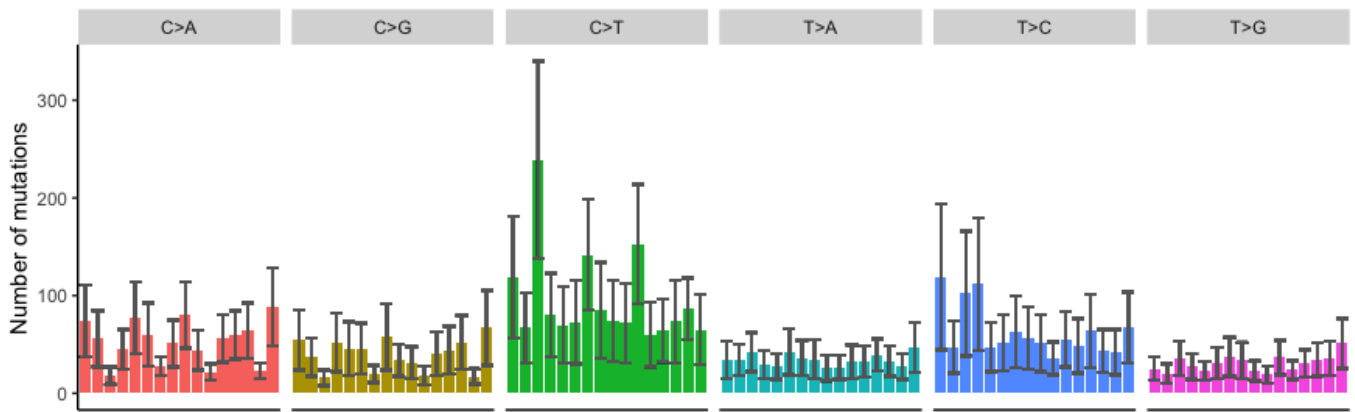**B** True signatures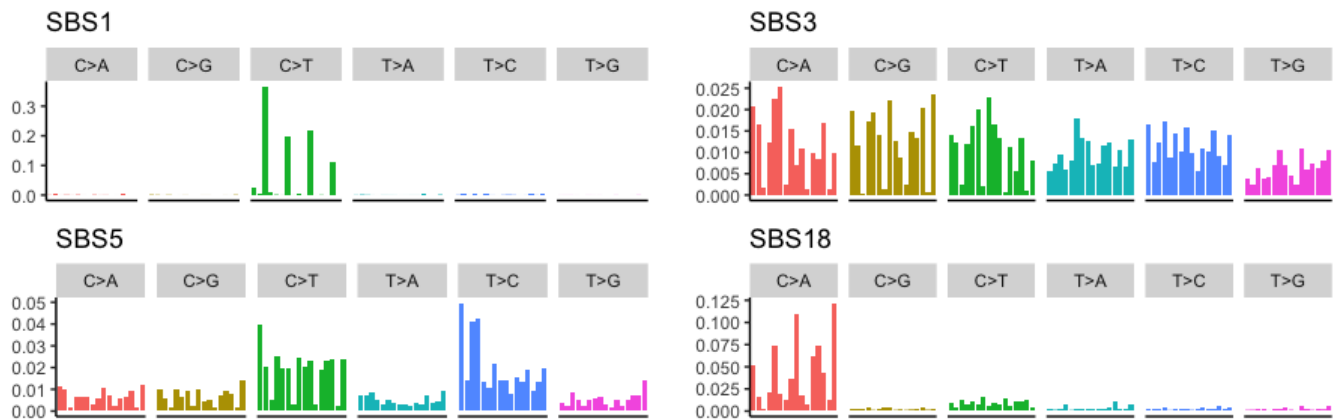**C** SparseSignatures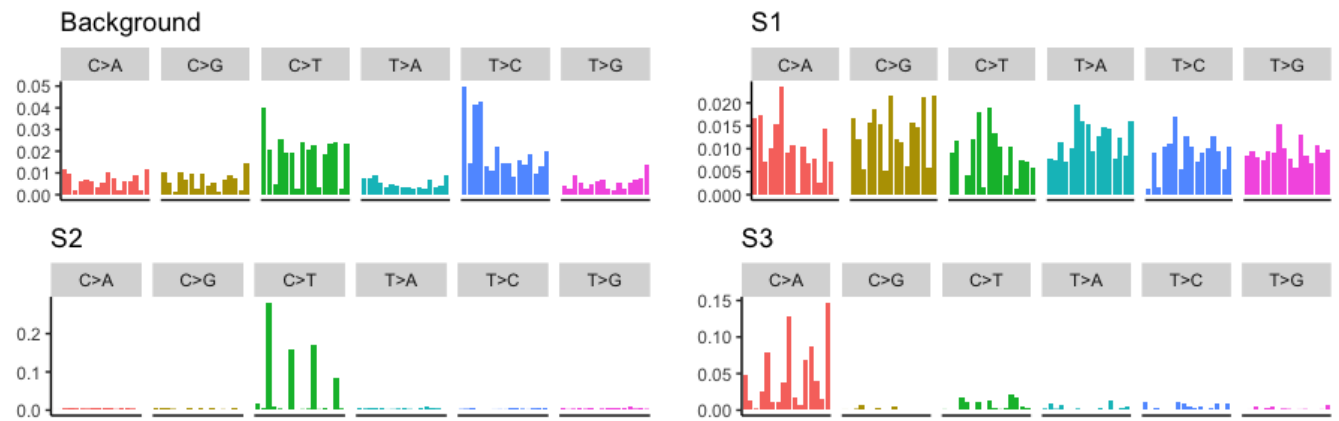**D** SparseSignatures - no background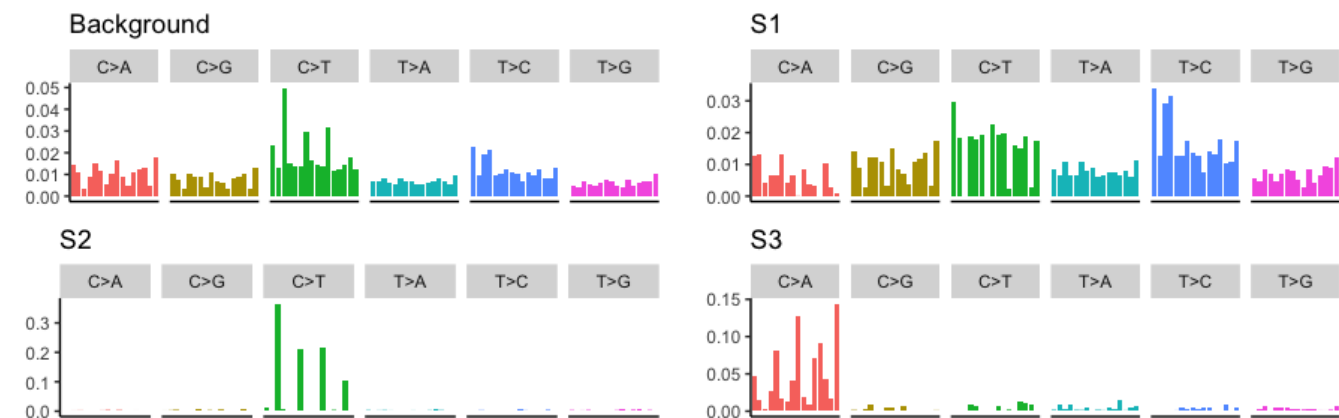

Supplement: S1 Fig — A) Average mutational counts for 116 simulated patients in each of 96 mutational categories. This dataset is one of 50 datasets simulated as part of Simulation 1. Error bars represent standard deviation. B) 4 original signatures in the simulated dataset. C) 4 signatures deciphered by SparseSignatures from the simulated dataset. D) 4 signatures deciphered by SparseSignatures from the simulated dataset, without the fixed background. Source data are provided in S3 Table. (PDF) [file pcbi.1009119.s002.pdf]

## A SigProfiler

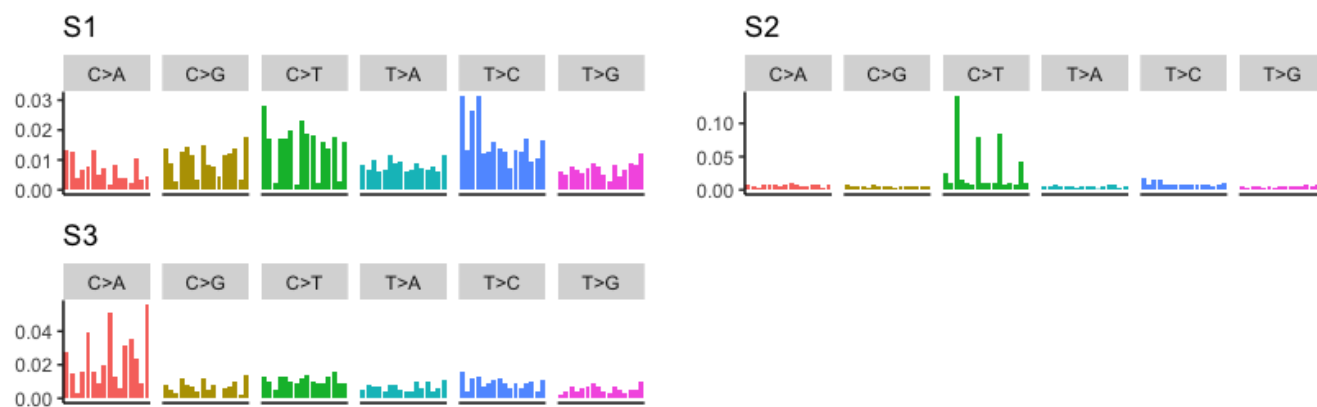

## B SignatureAnalyzer

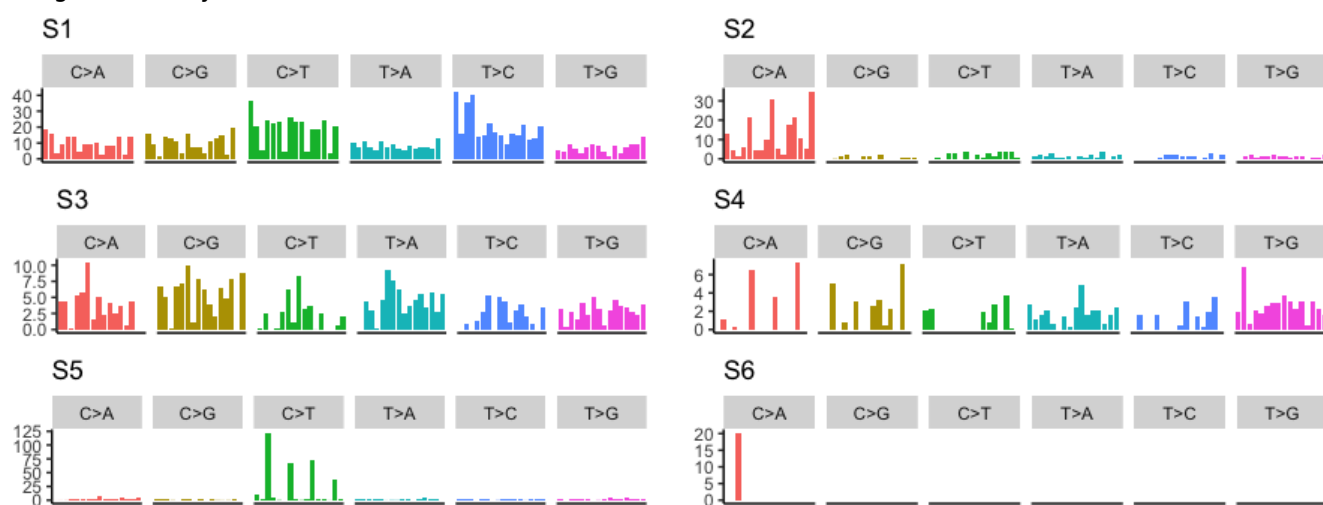

## C signer

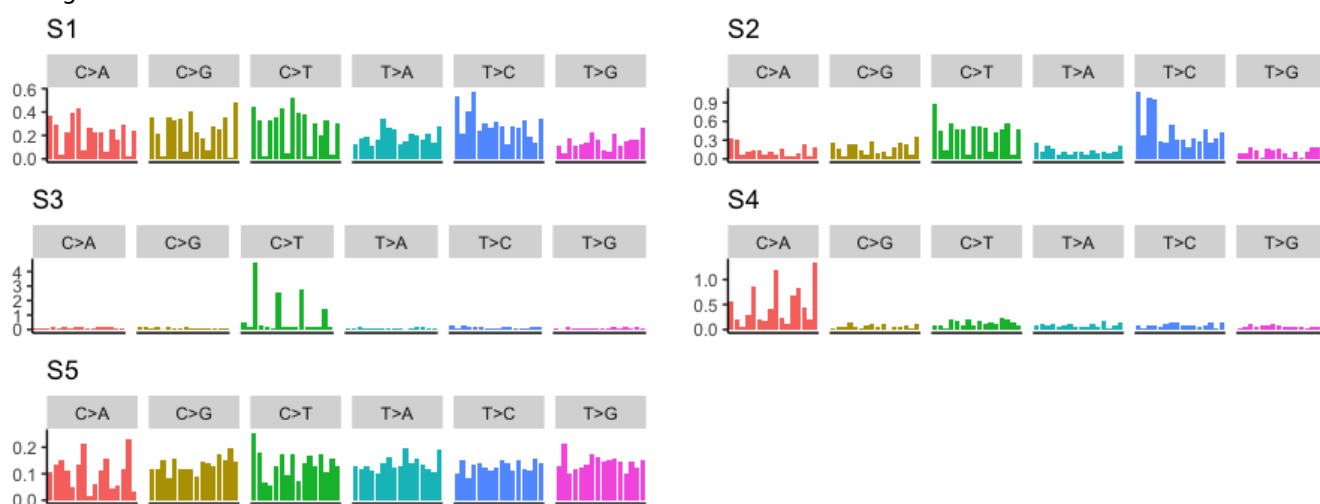

Supplement: S2 Fig — A) 4 signatures deciphered by SigProfiler from the simulated dataset shown in S1A Fig. B) 4 signatures deciphered by SignatureAnalyzer from the simulated dataset. C) 4 signatures deciphered by signeR from the simulated dataset. Source data are provided in S3 Table. (PDF) [file pcbi.1009119.s003.pdf]

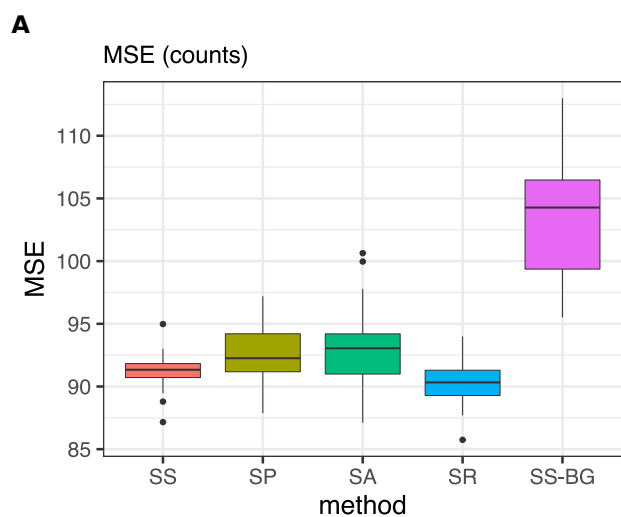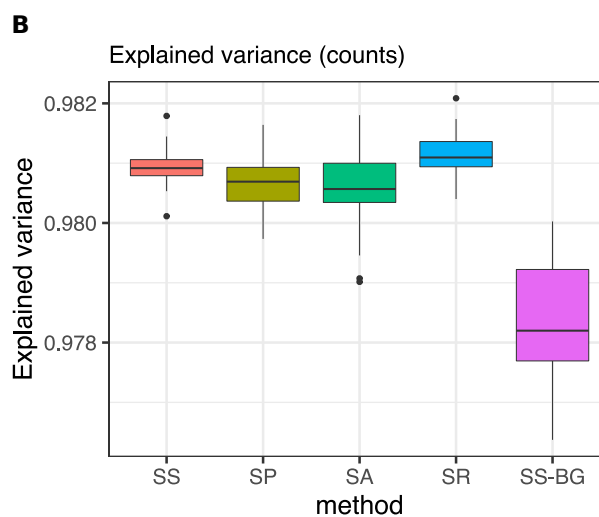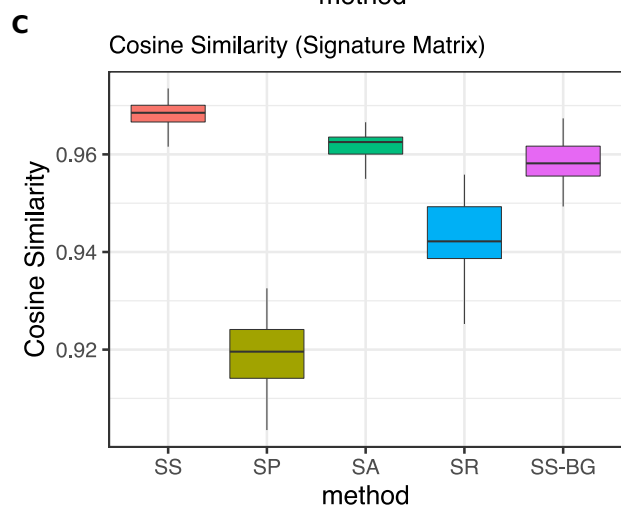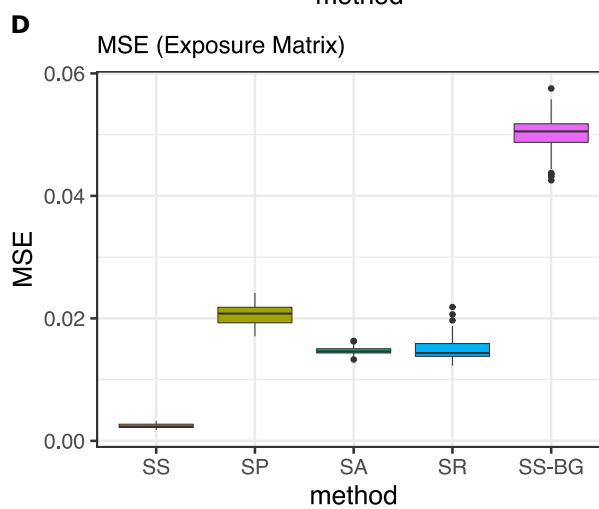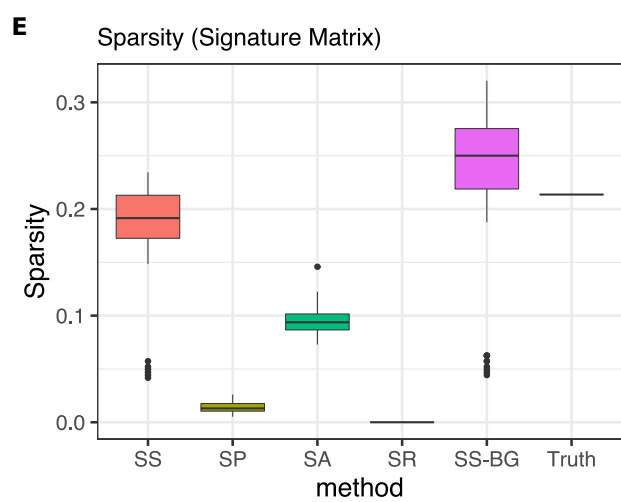

Supplement: S3 Fig — A) Box plots showing the residual error for the solutions produced by each method, over 50 simulations. Residual error was measured as the mean squared error (MSE) in reconstructing the original count matrix. B) Box plots showing the fraction of variance in the count matrix explained by the solutions produced by each method, over 50 simulations. (C) Box plots showing the cosine similarity of reconstructing the 3 non-background input signatures, over 50 simulations. D) Box plots showing the mean squared error in reconstructing the exposure values for the 3 non-background input signatures, over 50 simulations. E) Box plots showing the sparsity of the signatures produced by each method, over 50 simulations. Sparsity was measured as the fraction of cells in the signature matrix whose value is <10−3. SS: SparseSignatures. SP: SigProfiler. SA: SignatureAnalyzer. SR: signeR. Source data are provided in S4 Table. (PDF) [file pcbi.1009119.s004.pdf]

**A**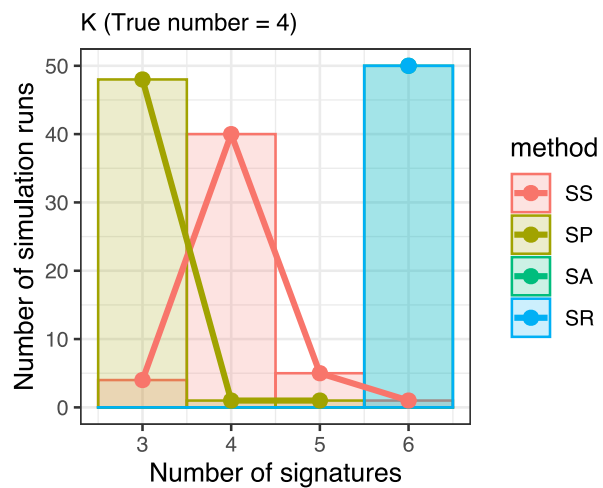**B**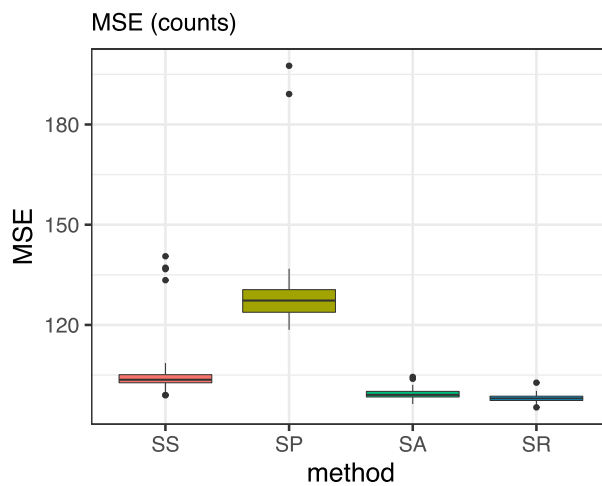**C**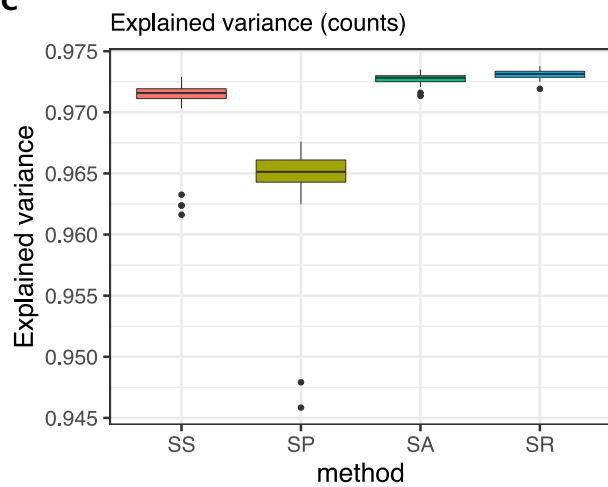**D**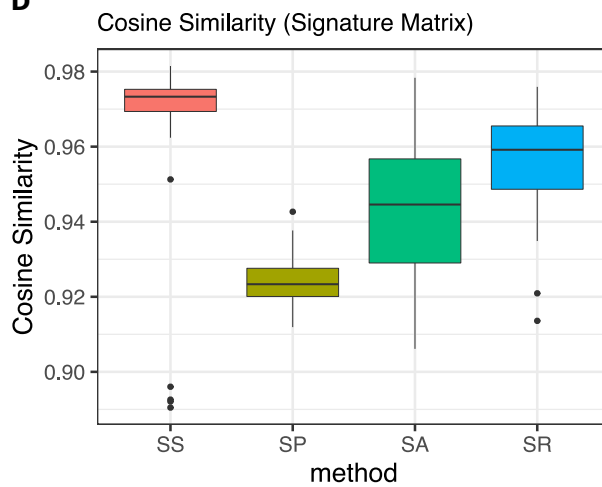**E**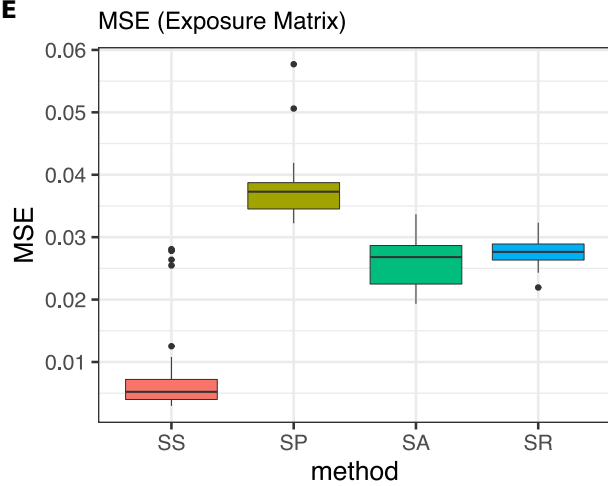**F**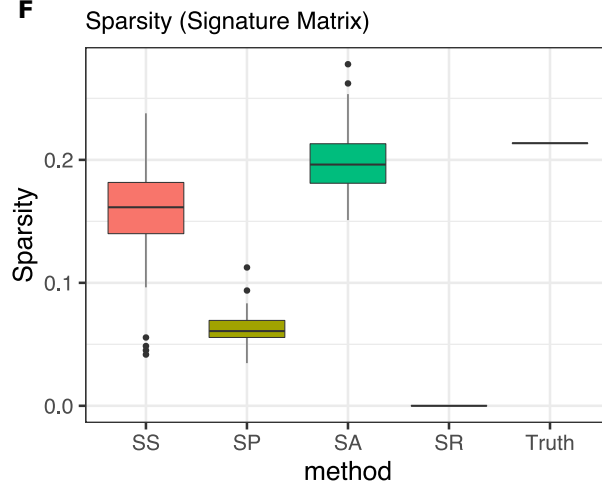

Supplement: S4 Fig — A) Bar and line plot showing, for each method, the number of simulation runs in which it selected each value of K (number of signatures). The x-axis shows values of K and the y-axis shows the number of times each value was selected. Each method was run on 50 simulated datasets. In all cases, the correct value of K was 4. B) Box plots showing the residual error for the solutions produced by each method, over 50 simulations. Residual error was measured as the mean squared error (MSE) in reconstructing the original count matrix. C) Box plots showing the fraction of variance in the count matrix explained by the solutions produced by each method, over 50 simulations. D) Box plots showing the cosine similarity of reconstructing the 3 non-background input signatures, over 50 simulations. E) Box plots showing the mean squared error in reconstructing the exposure values for the 3 non-background input signatures, over 50 simulations. F) Box plots showing the sparsity of the signatures produced by each method, over 50 simulations. Sparsity was measured as the fraction of cells in the signature matrix whose value is <10−3. SS: SparseSignatures. SP: SigProfiler. SA: SignatureAnalyzer. SR: signeR. Source data are provided in S5 Table. (PDF) [file pcbi.1009119.s005.pdf]

**A**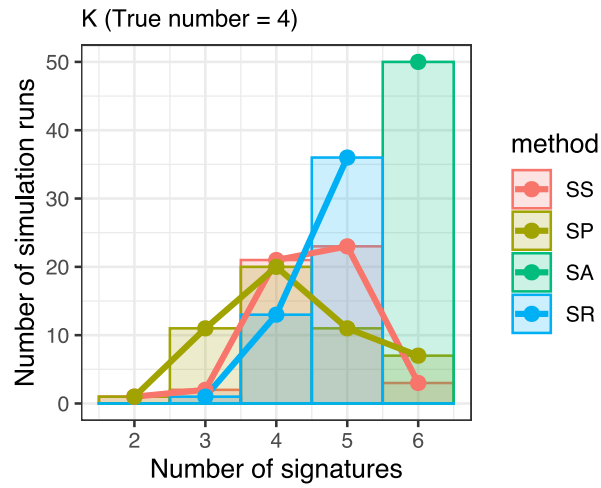**B**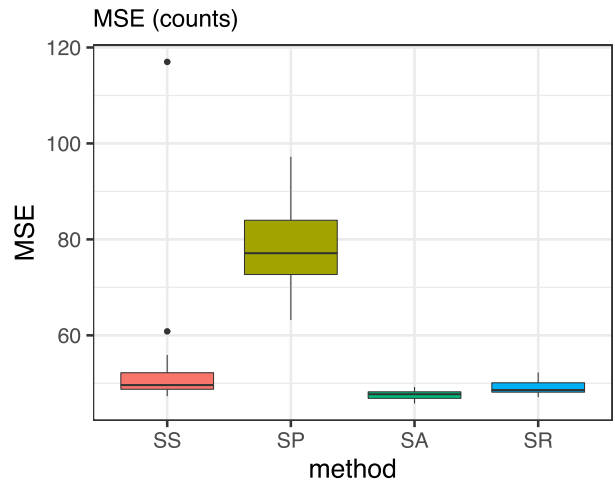**C**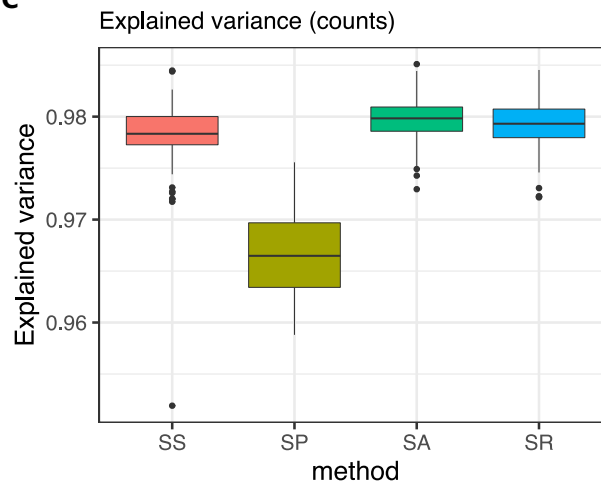**D**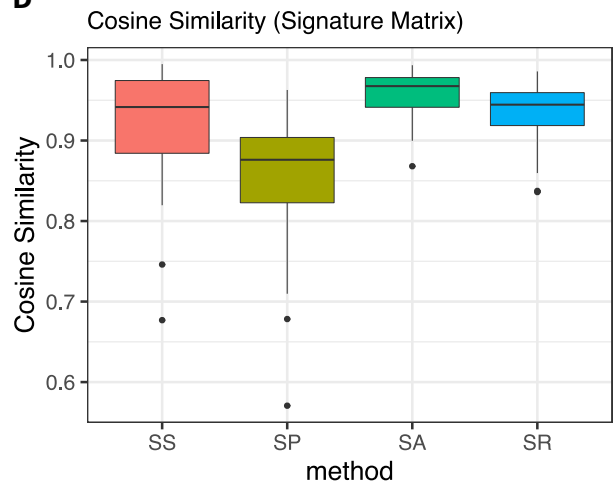**E**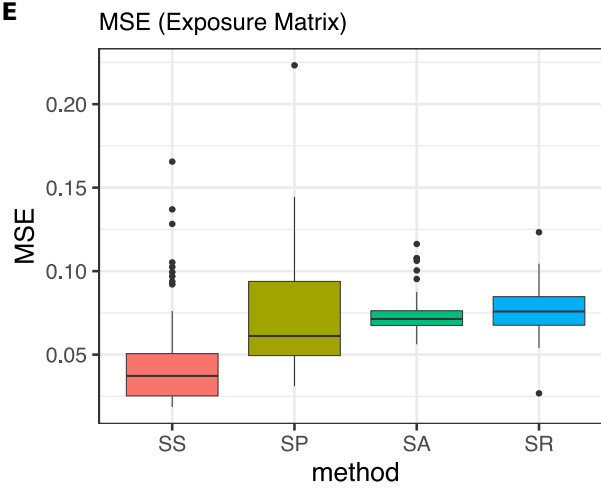**F**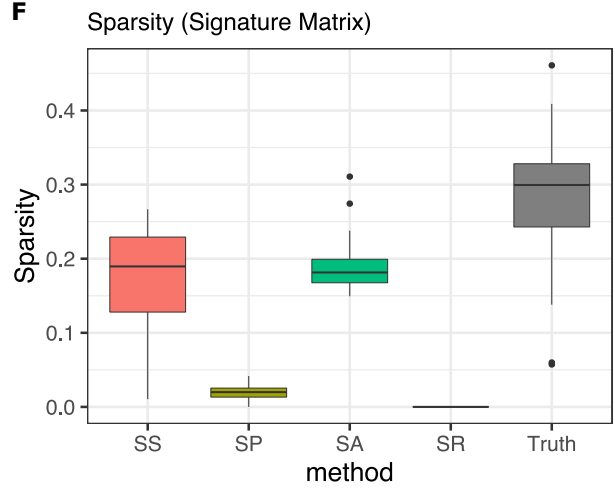

Supplement: S5 Fig — A) Bar and line plot showing, for each method, the number of simulation runs in which it selected each value of K (number of signatures). The x-axis shows values of K and the y-axis shows the number of times each value was selected. Each method was run on 50 simulated datasets. In all cases, the correct value of K was 4. B) Box plots showing the residual error for the solutions produced by each method, over 50 simulations. Residual error was measured as the mean squared error (MSE) in reconstructing the original count matrix. C) Box plots showing the fraction of variance in the count matrix explained by the solutions produced by each method, over 50 simulations. D) Box plots showing the cosine similarity of reconstructing the 3 non-background input signatures, over 50 simulations. E) Box plots showing the mean squared error in reconstructing the exposure values for the 3 non-background input signatures, over 50 simulations. F) Box plots showing the sparsity of the signatures produced by each method, over 50 simulations. Sparsity was measured as the fraction of cells in the signature matrix whose value is <10−3. SS: SparseSignatures. SP: SigProfiler. SA: SignatureAnalyzer. SR: signeR. Source data are provided in S6 Table. (PDF) [file pcbi.1009119.s006.pdf]

**A**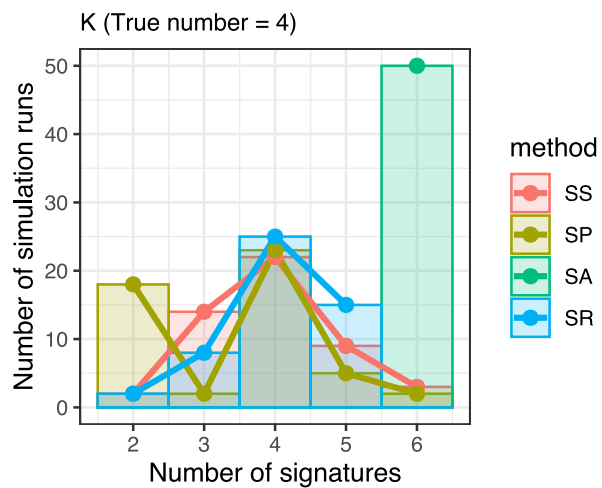**B**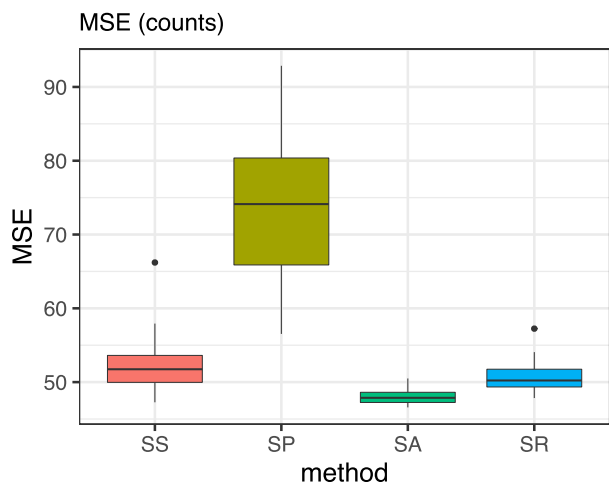**C**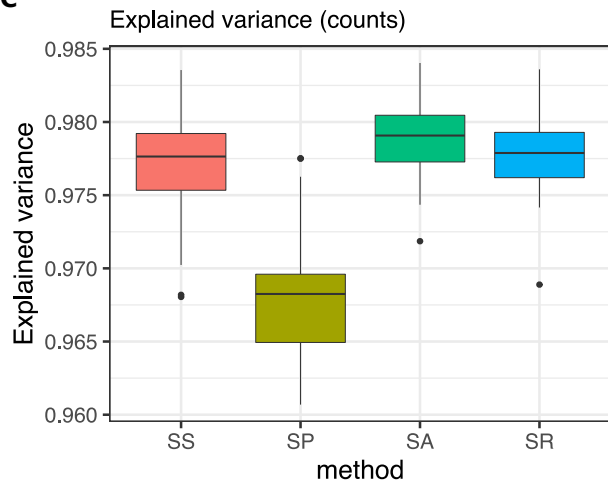**D**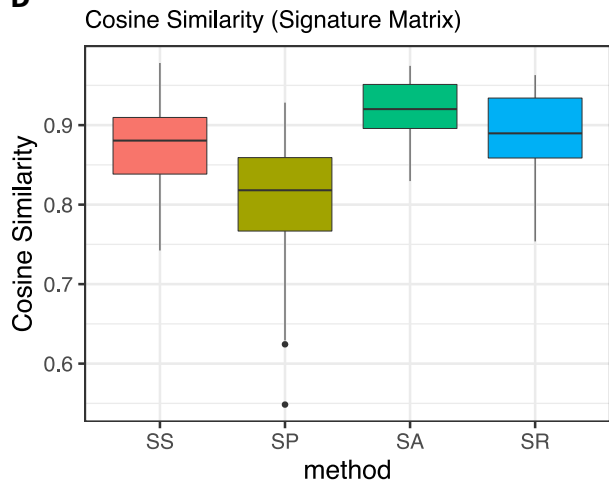**E**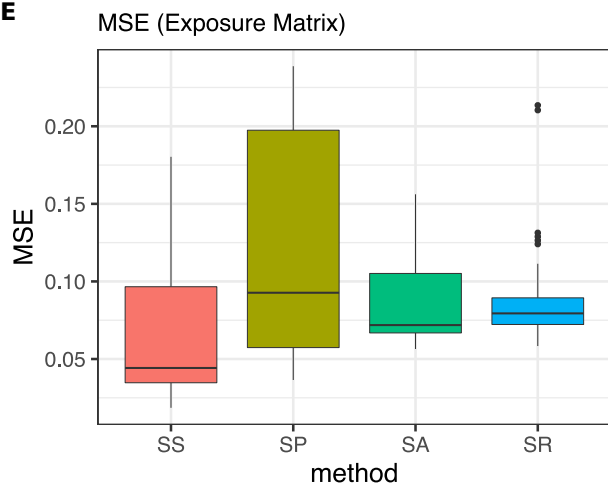**F**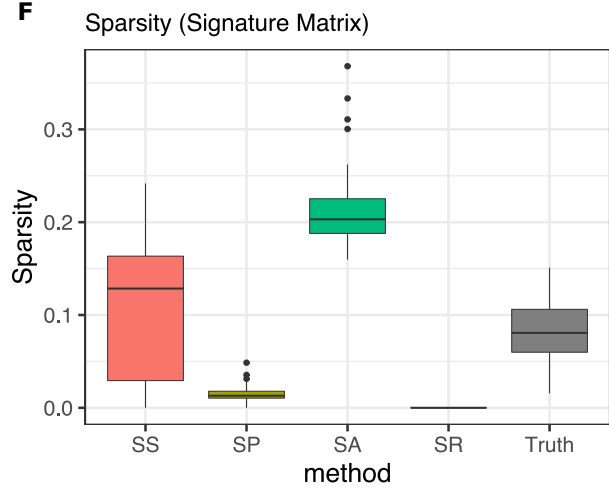

Supplement: S6 Fig — A) Bar and line plot showing, for each method, the number of simulations in which it selected each value of K (number of signatures). The x-axis shows values of K and the y-axis shows the number of times each value was selected. Each method was run on 50 simulated datasets. In all cases, the correct value of K was 4. B) Box plots showing the residual error for the solutions produced by each method, over 50 simulations. Residual error was measured as the mean squared error (MSE) in reconstructing the original count matrix. C) Box plots showing the fraction of variance in the count matrix explained by the solutions produced by each method, over 50 simulations. D) Box plots showing the mean squared error in reconstructing the 3 non-background input signatures, over 50 simulations. E) Box plots showing the mean squared error in reconstructing the exposure values for the 3 non-background input signatures, over 50 simulations. F) Box plots showing the sparsity of the signatures produced by each method, over 50 simulations. Sparsity was measured as the fraction of cells in the signature matrix whose value is <10−3. SS: SparseSignatures. SP: SigProfiler. SA: SignatureAnalyzer. SR: signeR. Source data are provided in S7 Table. (PDF) [file pcbi.1009119.s007.pdf]

**A**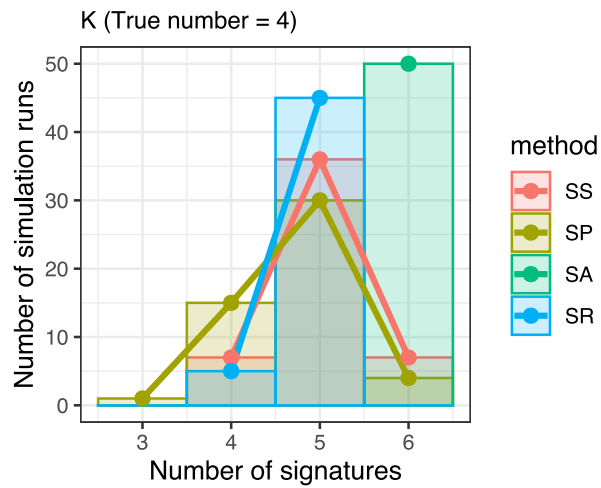**B**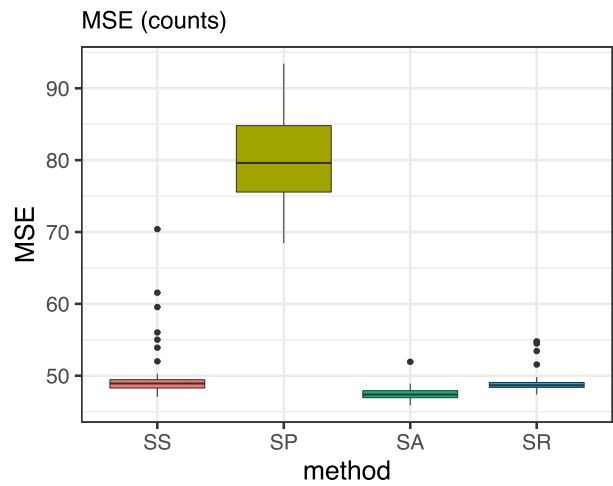**C**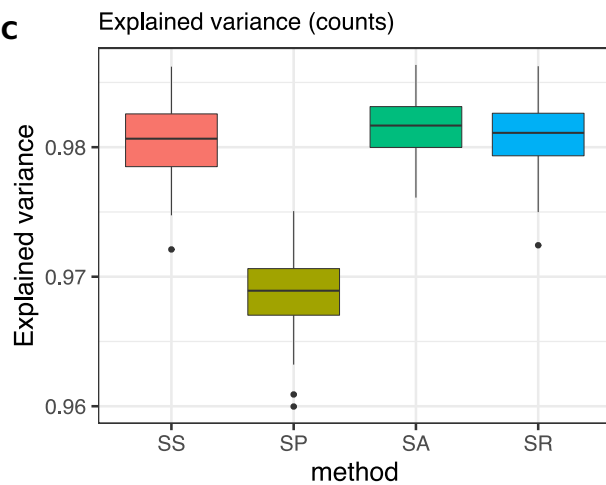**D**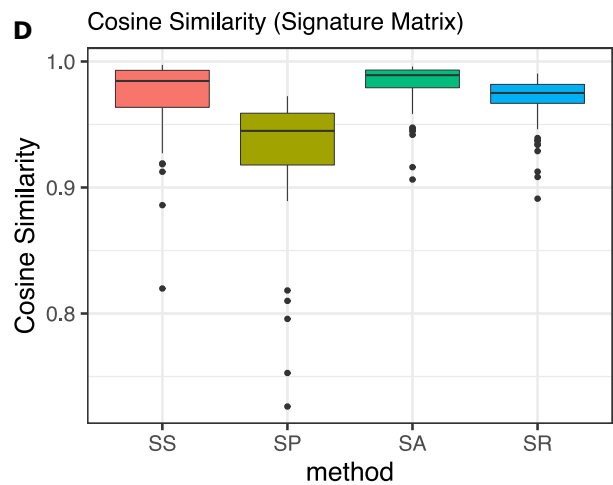**E**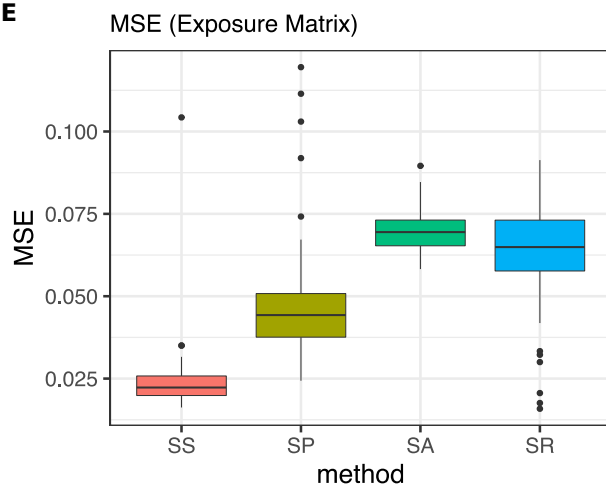**F**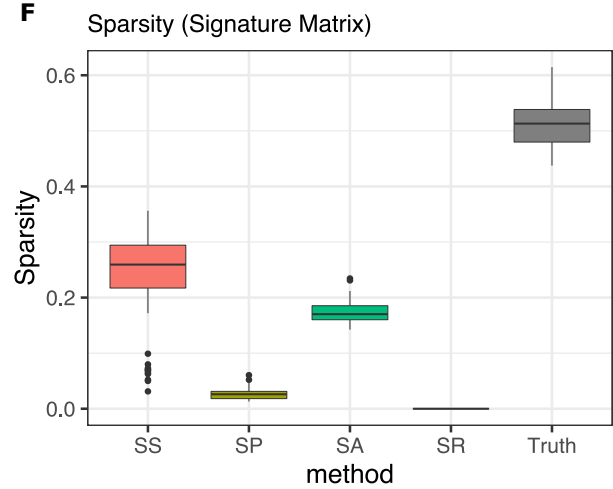

Supplement: S7 Fig — A) Bar and line plot showing, for each method, the number of simulations in which it selected each value of K (number of signatures). The x-axis shows values of K and the y-axis shows the number of times each value was selected. Each method was run on 50 simulated datasets. In all cases, the correct value of K was 4. B) Box plots showing the residual error for the solutions produced by each method, over 50 simulations. Residual error was measured as the mean squared error (MSE) in reconstructing the original count matrix. C) Box plots showing the fraction of variance in the count matrix explained by the solutions produced by each method, over 50 simulations. D) Box plots showing the cosine similarity of reconstructing the 3 non-background input signatures, over 50 simulations. E) Box plots showing the mean squared error in reconstructing the exposure values for the 3 non-background input signatures, over 50 simulations. F) Box plots showing the sparsity of the signatures produced by each method, over 50 simulations. Sparsity was measured as the fraction of cells in the signature matrix whose value is <10−3. SS: SparseSignatures. SP: SigProfiler. SA: SignatureAnalyzer. SR: signeR. Source data are provided in S8 Table. (PDF) [file pcbi.1009119.s008.pdf]

**A**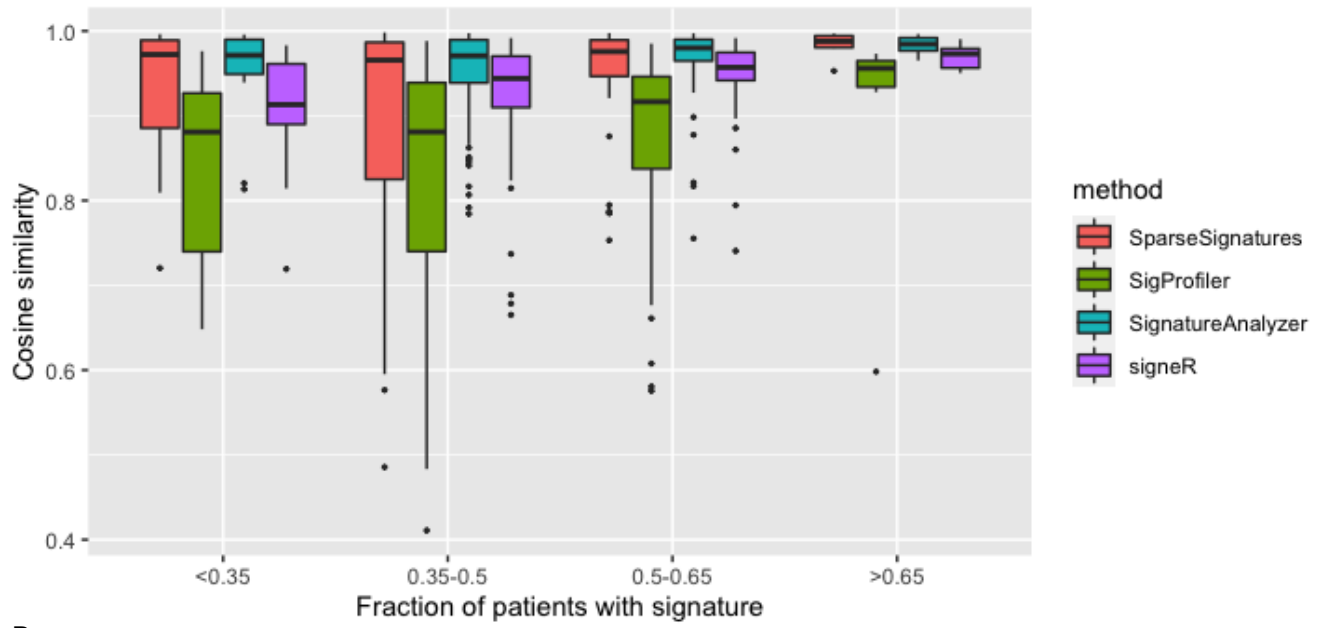**B**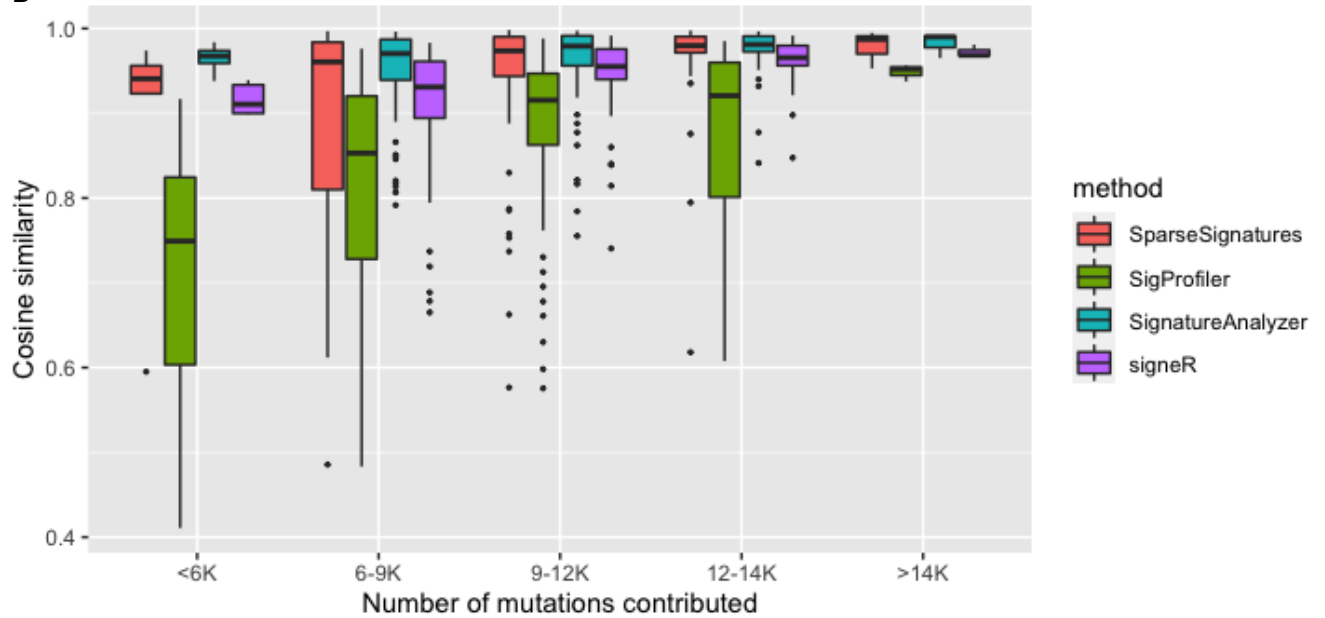

Supplement: S9 Fig — A) Boxplots showing the cosine similarity of signature reconstruction for signatures, separated by the fraction of patients in the population in which the signature is present. B) Boxplots showing the cosine similarity of signature reconstruction for signatures, separated by the number of mutations contributed by the signature in the overall dataset. Source data are provided in S10 Table. (PDF) [file pcbi.1009119.s010.pdf]

SPR1 (SBS1 - 0.99)

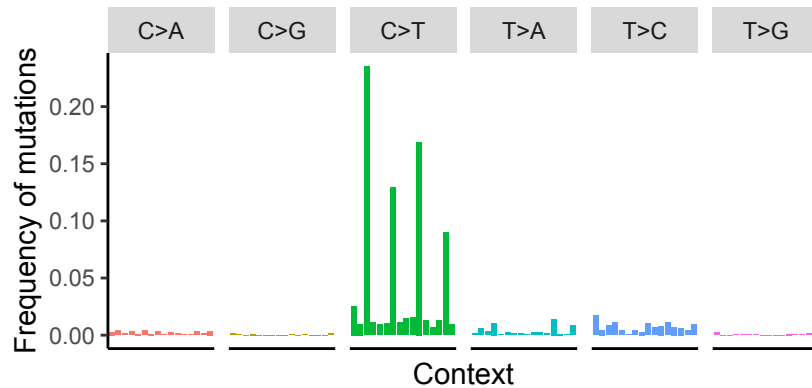

SPR2 (SBS2+13 - 0.96)

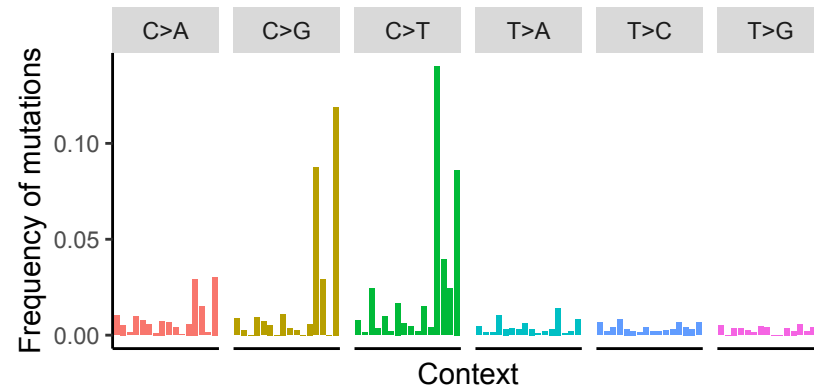

SPR3 (SBS3 - 0.93)

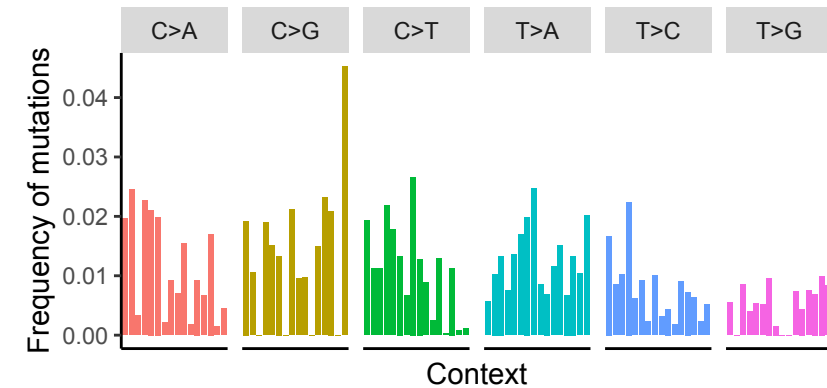

SPR4 (SBS17a+b - 0.86)

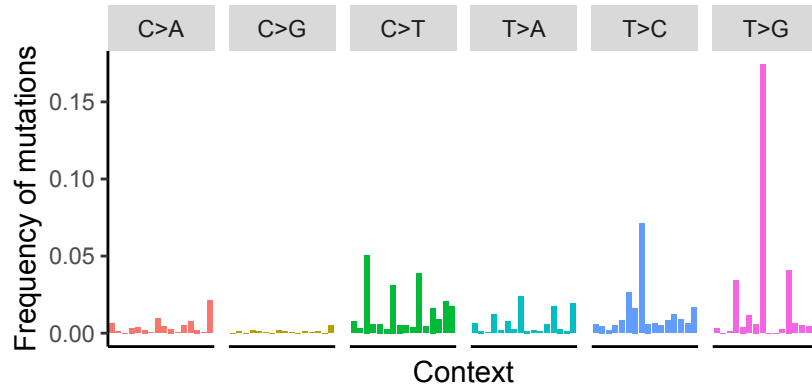

SPR5 (SBS18 - 0.86)

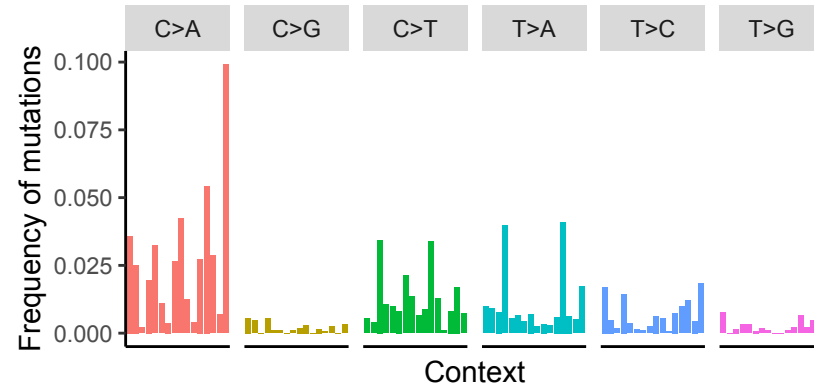

SPR6 (SBS26 - 0.88)

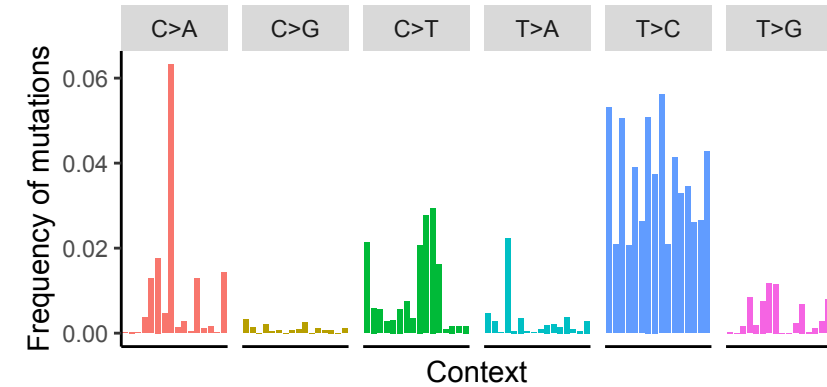

SPR7 (SBS30 - 0.82)

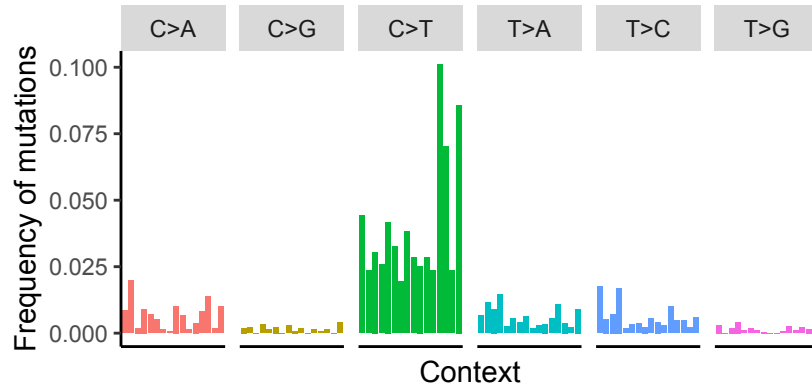

SPR8 (SBS51 - 0.88)

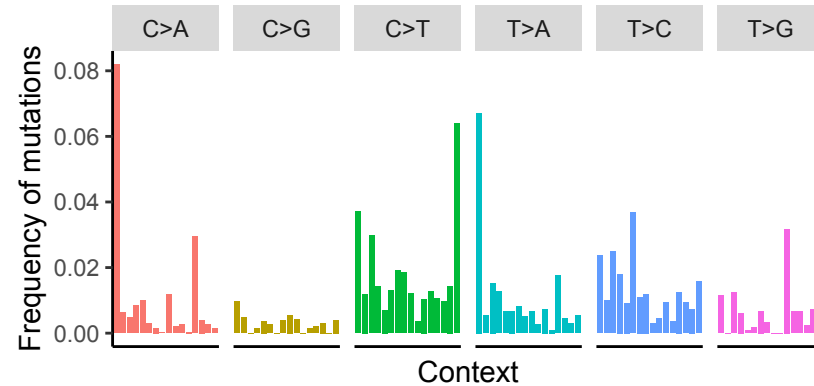

Supplement: S10 Fig — Source data are provided in S15 Table. (PDF) [file pcbi.1009119.s011.pdf]

SIA1 (SBS1 - 0.98)

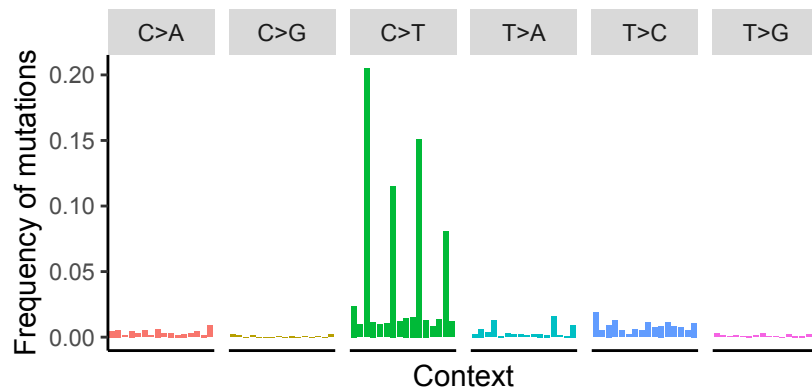

SIA2 (SBS2+13 - 1.00)

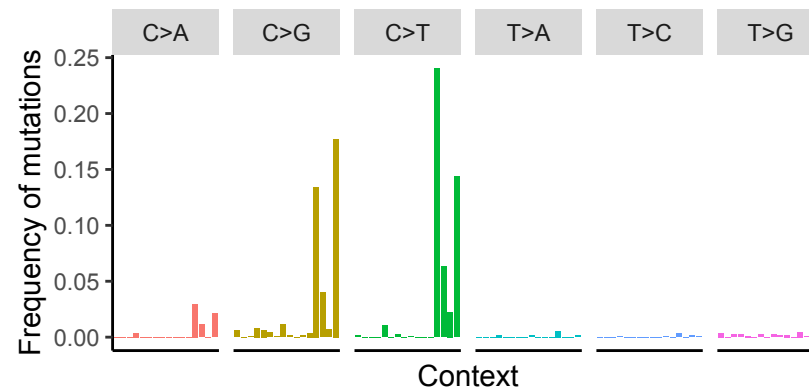

SIA3 (SBS3 - 0.91)

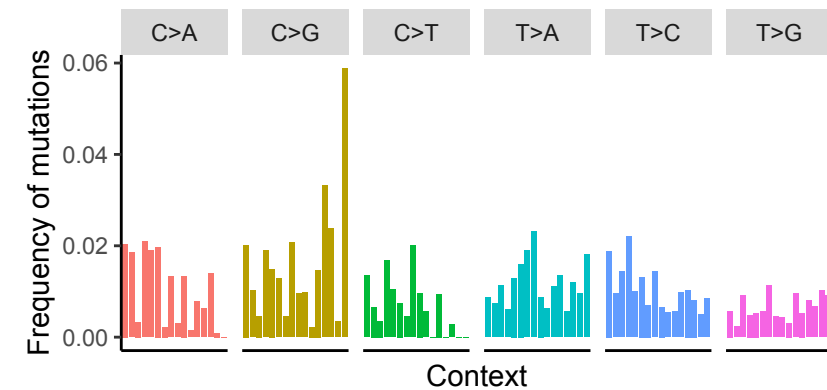

SIA4 (SBS17a+b - 0.93)

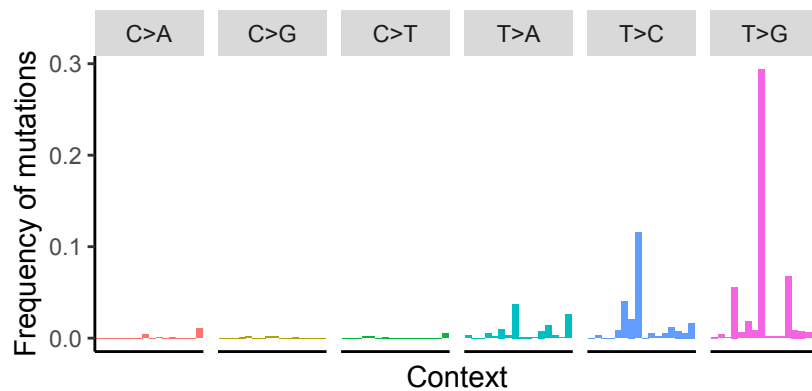

SIA5 (SBS18 - 0.89)

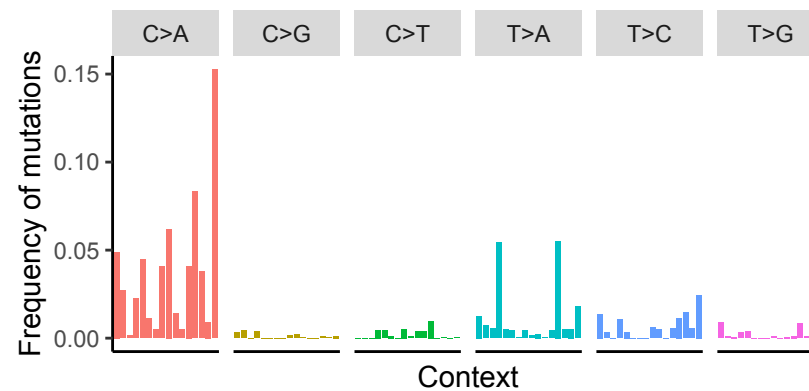

SIA6 (SBS26 - 0.89)

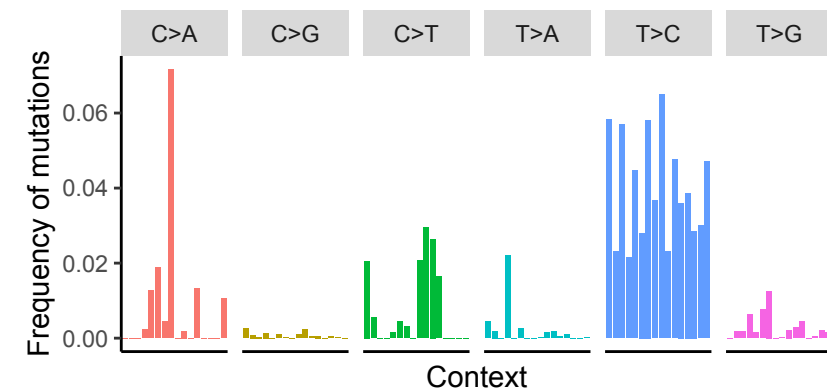

SIA7 (SBS30 - 0.81)

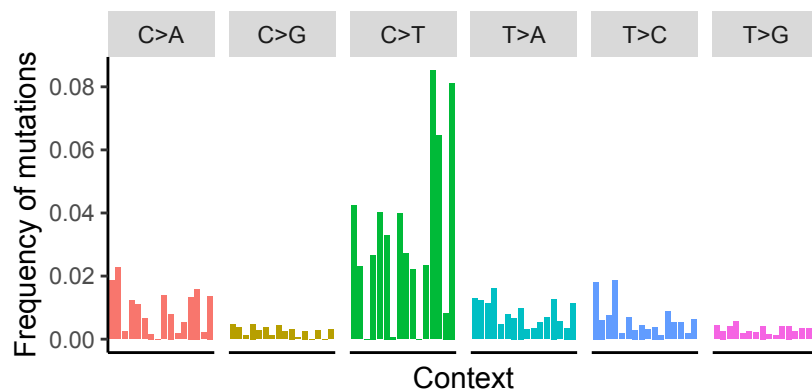

SIA8 (SBS51 - 0.93)

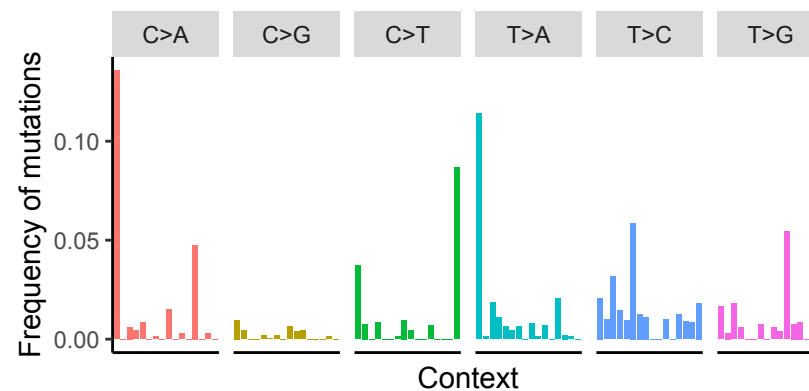

Supplement: S11 Fig — Source data are provided in S16 Table. (PDF) [file pcbi.1009119.s012.pdf]

SIP1 (SBS1 - 0.99)

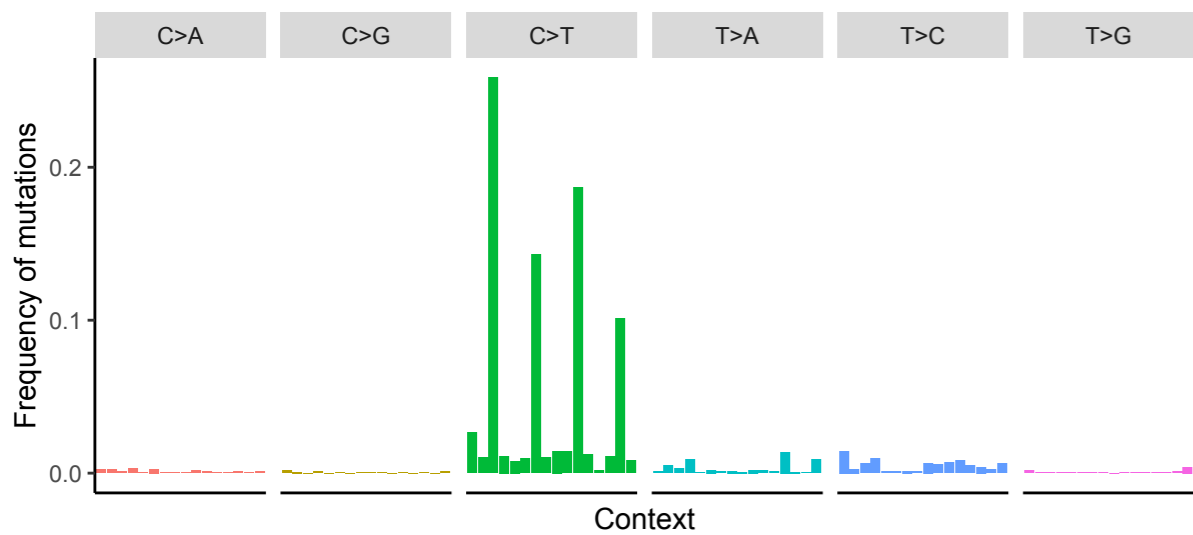

SIP2 (SBS2+13 - 0.99)

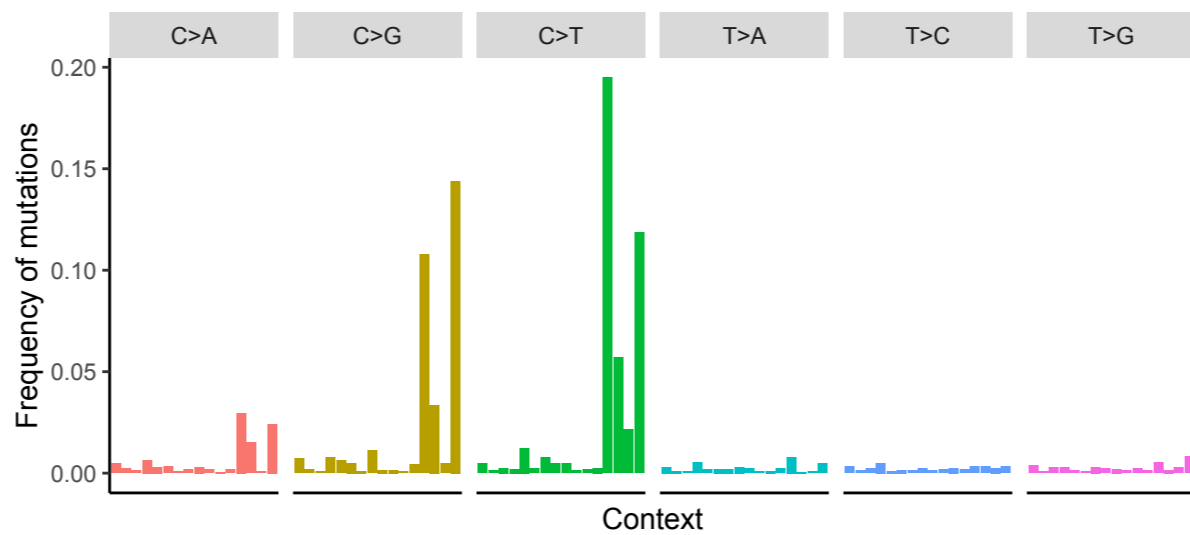

SIP3 (SBS3 - 0.74)

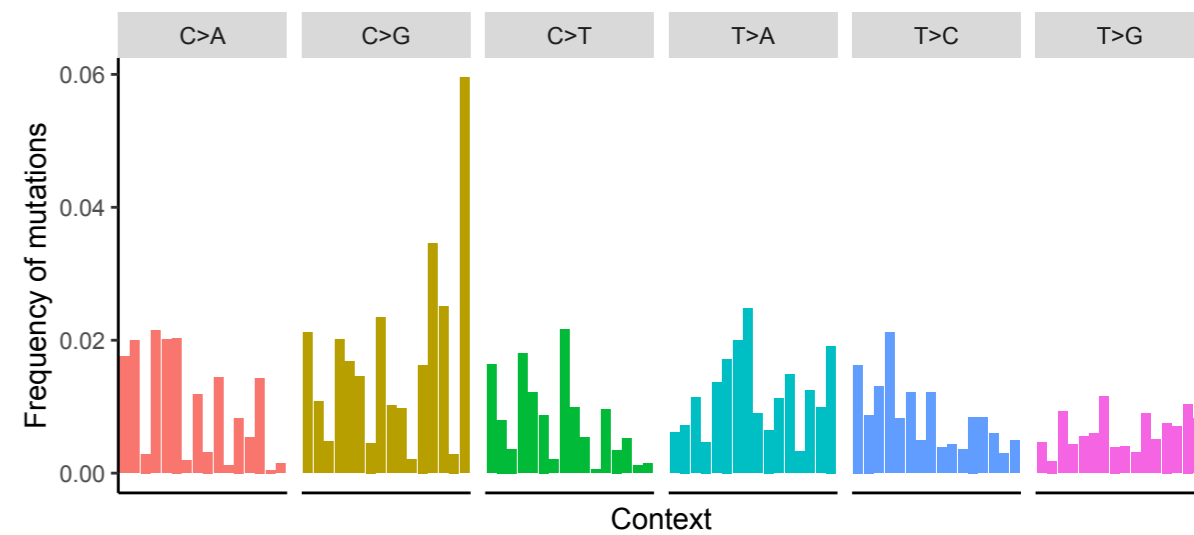

SIP4 (SBS17a+b - 0.91)

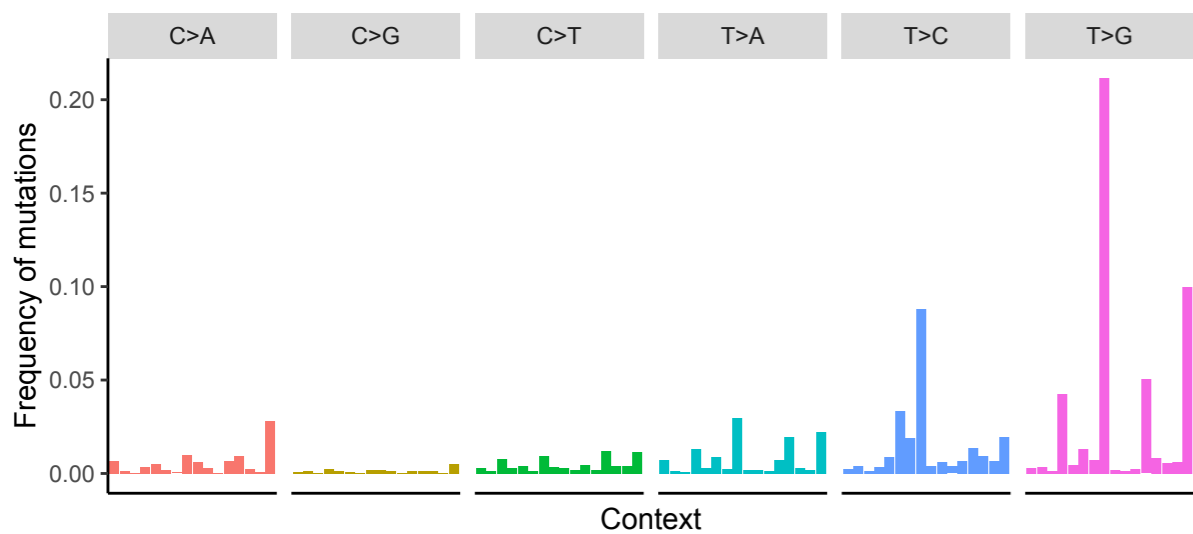

SIP5 (SBS18 - 0.66)

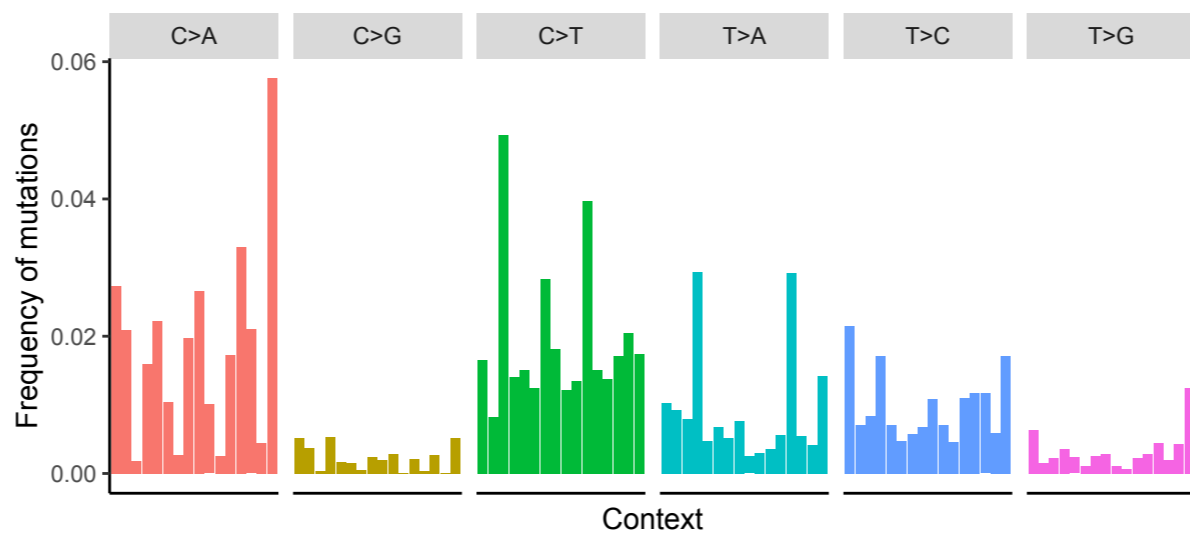

SIP6 (SBS26 - 0.85)

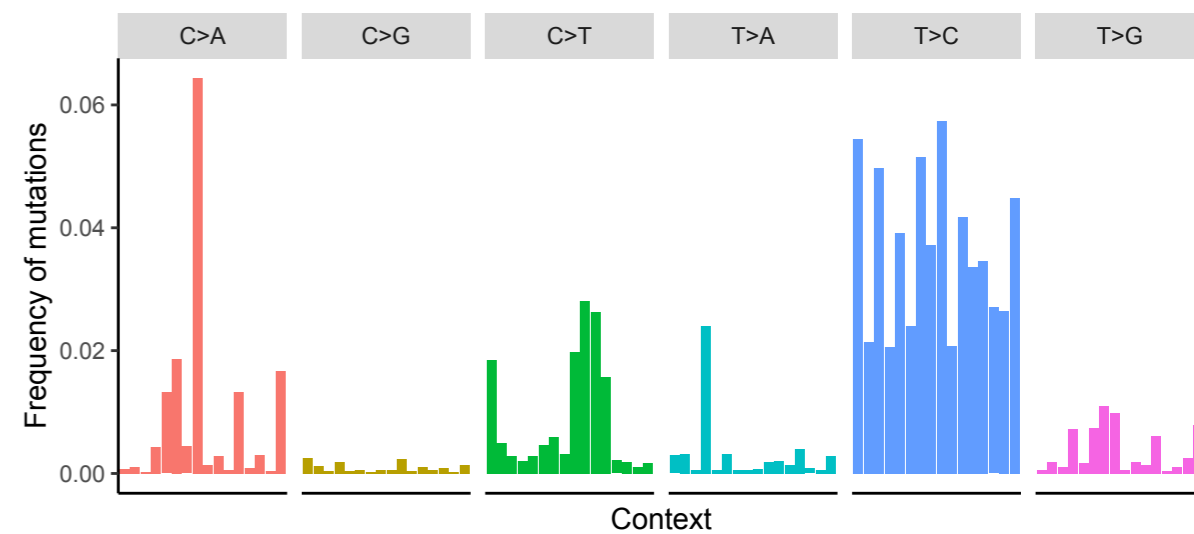

SIP7 (SBS30 - 0.81)

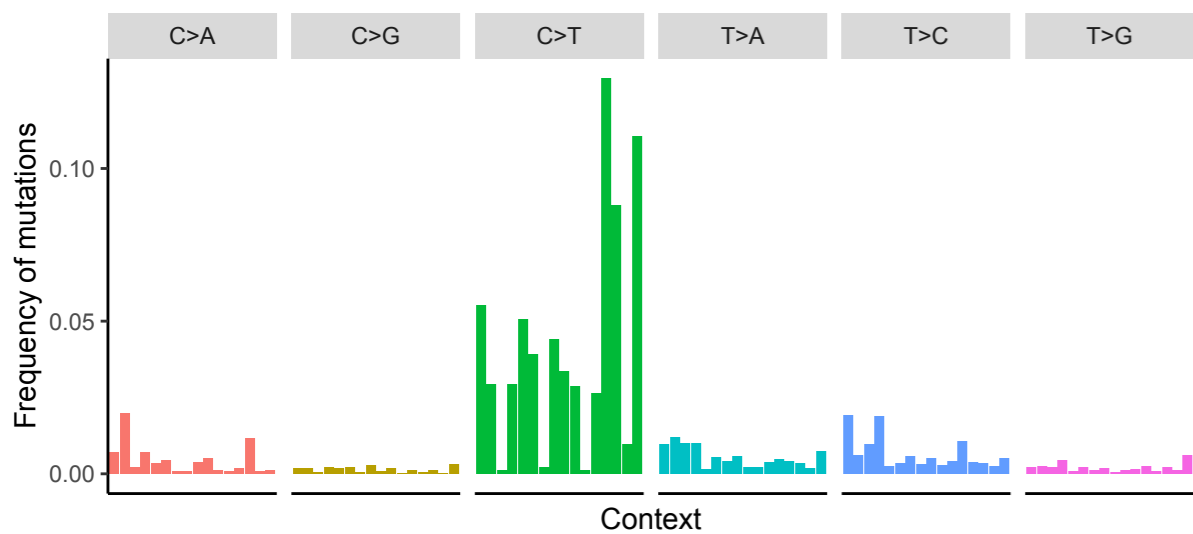

SIP8 (SBS51 - 0.89)

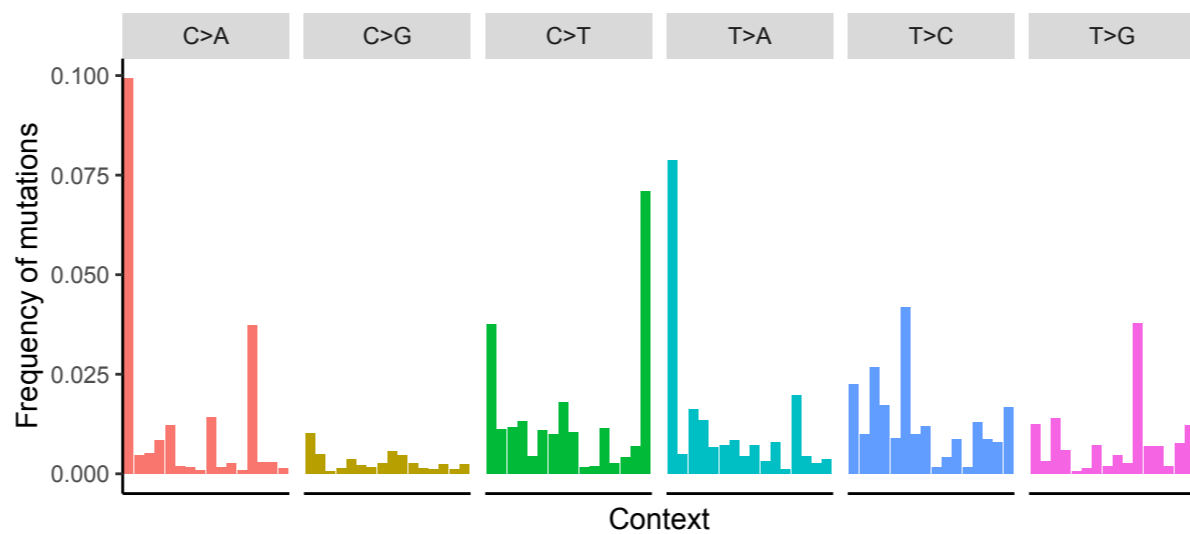

Supplement: S12 Fig — Source data are provided in S17 Table. (PDF) [file pcbi.1009119.s013.pdf]

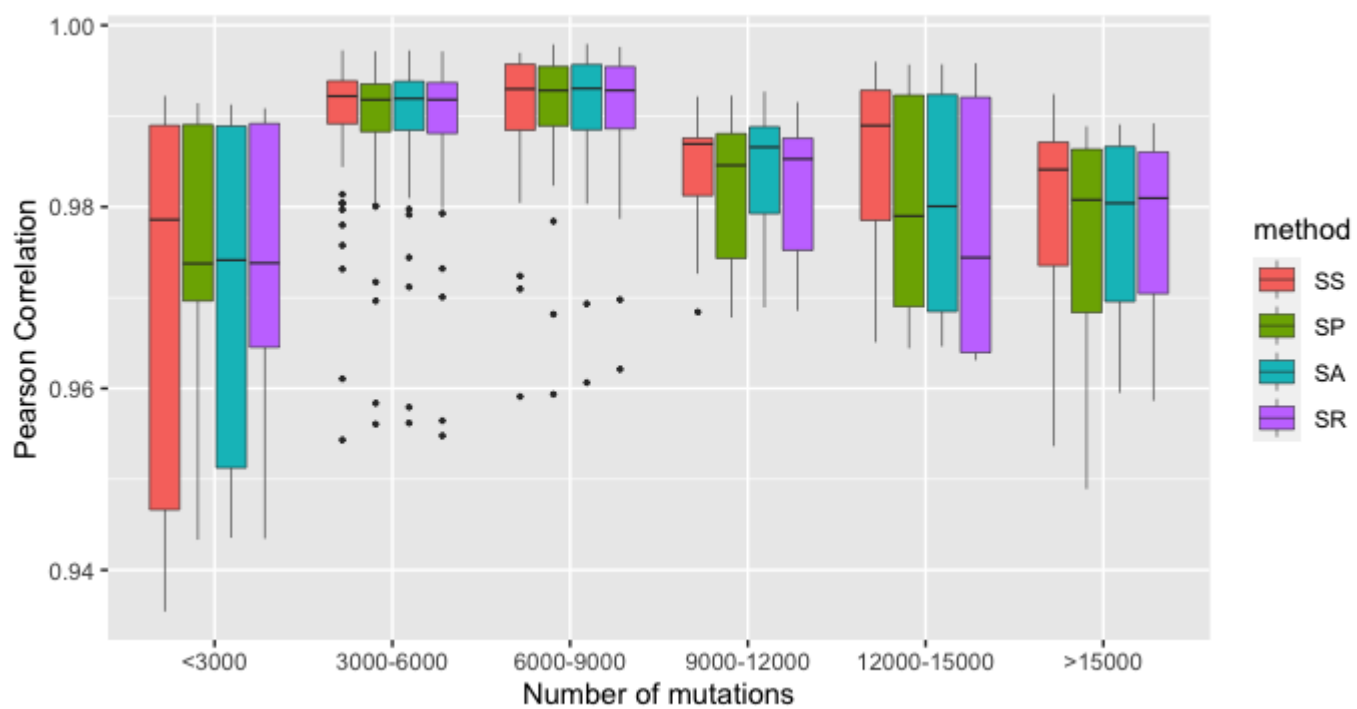

Supplement: S13 Fig — The x-axis shows the total number of mutations in the tumor. SS: SparseSignatures. SP: SigProfiler. SA: SignatureAnalyzer. SR: signeR. Source data are provided in S18 Table. (PDF) [file pcbi.1009119.s014.pdf]

**A**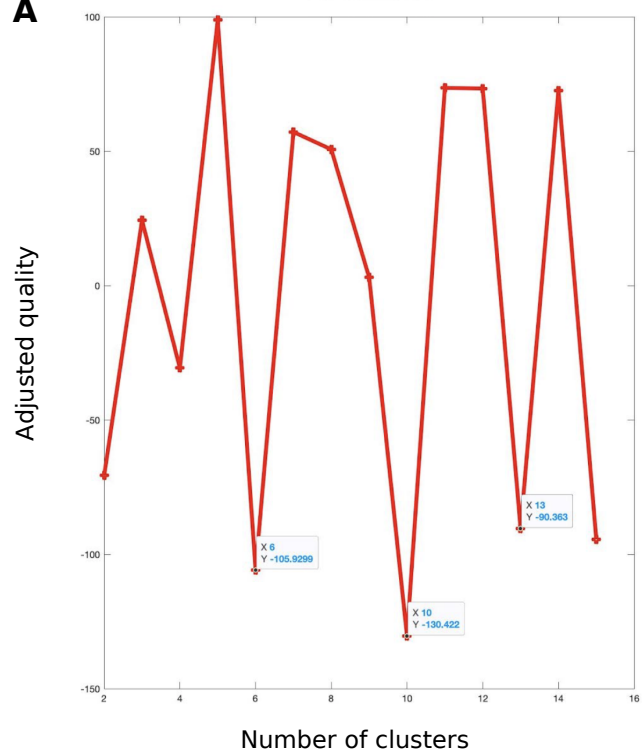**B**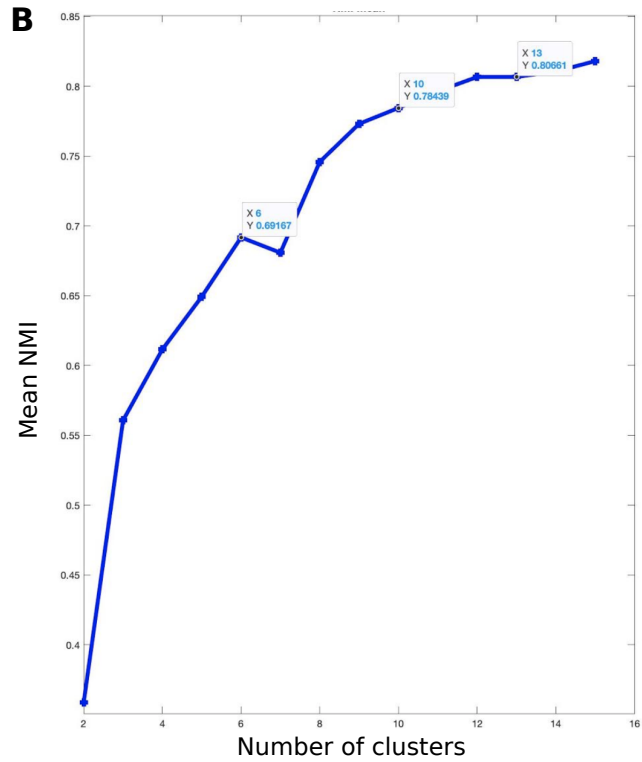

Supplement: S14 Fig — A) CIMLR was first run on the original dataset; then clustering was repeated 100 times on datasets generated by bootstrap resampling. The figure reports mean normalized mutual information (NMI) between cluster assignments across the bootstraps; higher values indicate stable results. B) CIMLR number of clusters for SparseSignatures. (PDF) [file pcbi.1009119.s015.pdf]

**A**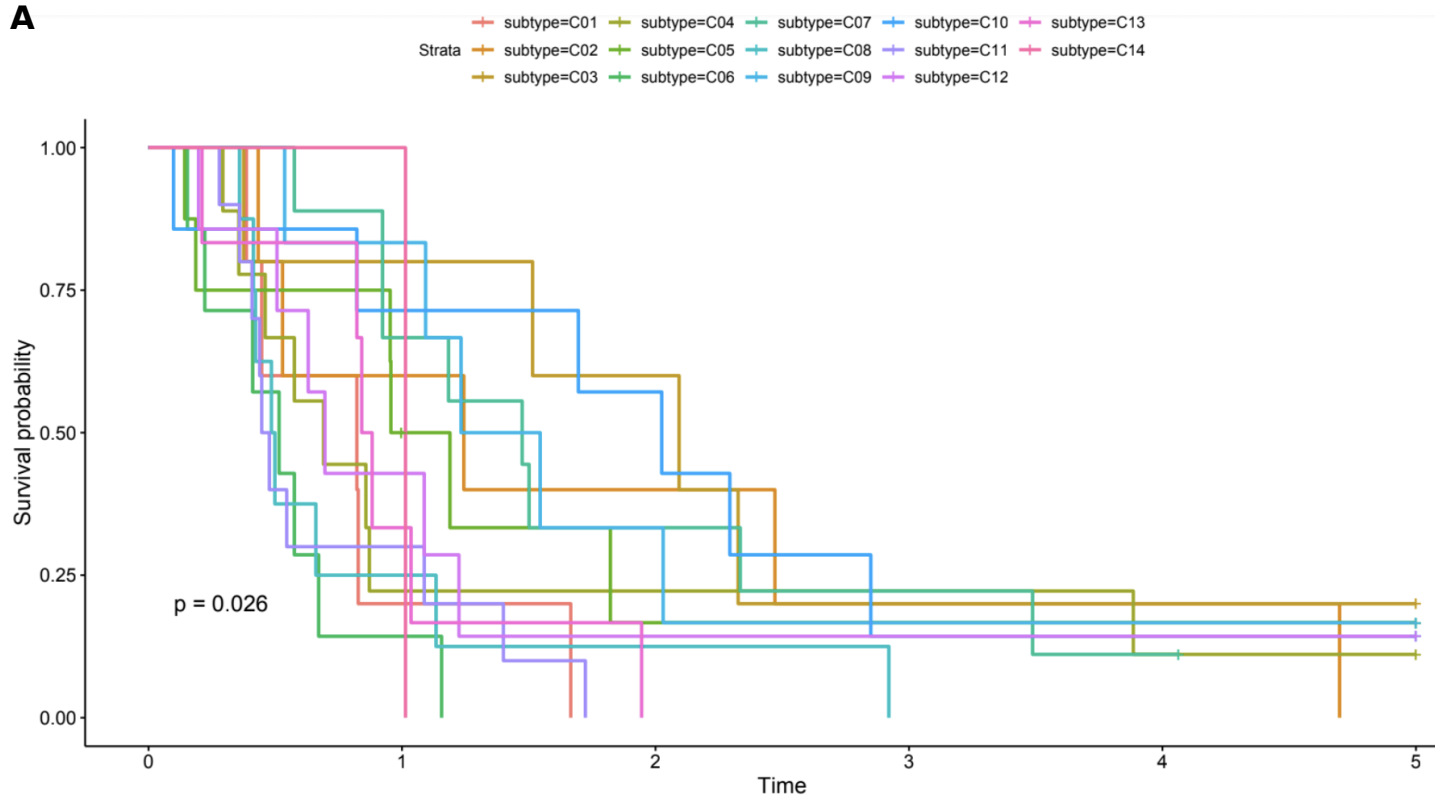**B**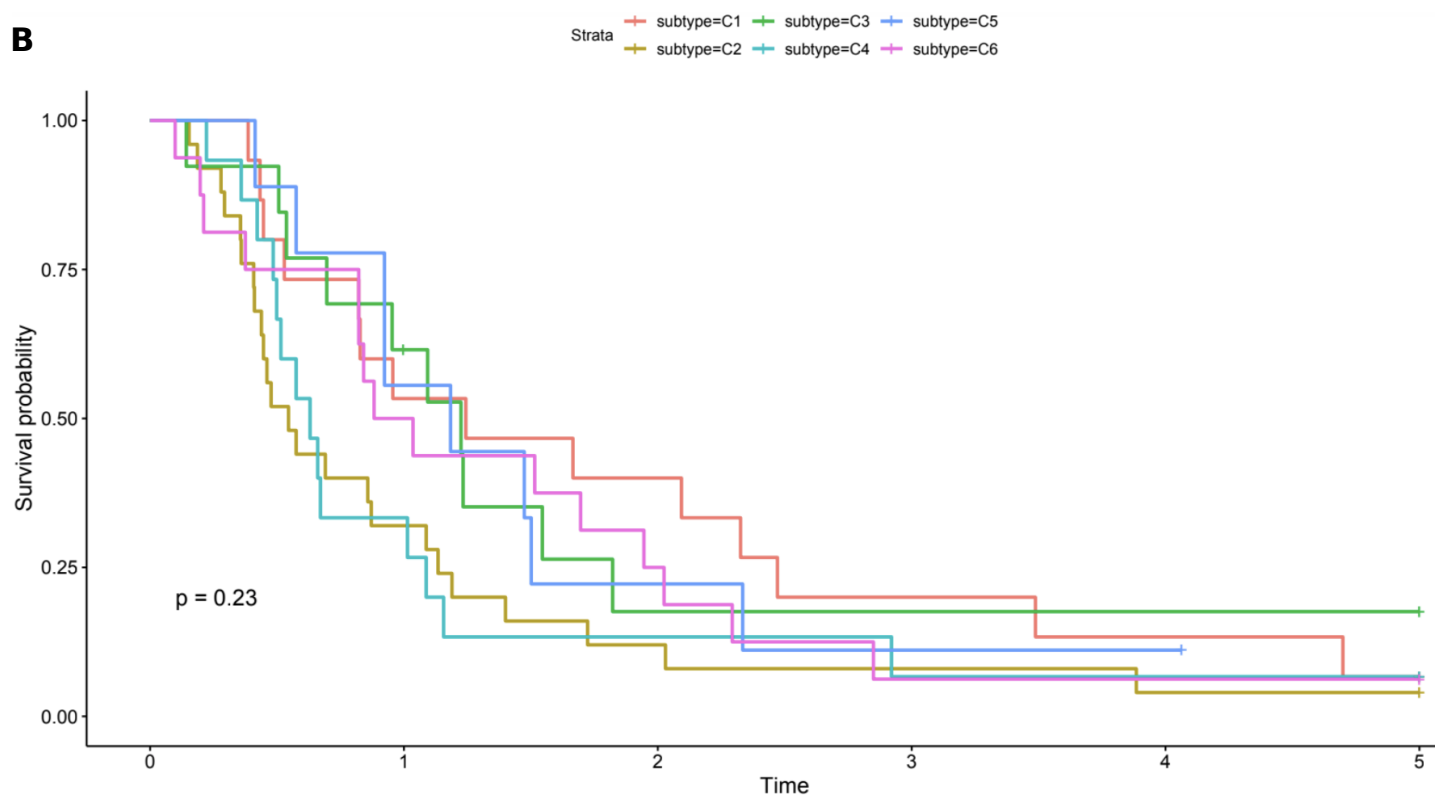

Supplement: S15 Fig — A) Survival curves for pancreatic cancer patients, divided into CIMLR clusters based on SigProfiler results. B) Survival curves for pancreatic cancer patients, divided into CIMLR clusters based on SignatureAnalyzer results. C) Survival curves for pancreatic cancer patients, divided into CIMLR clusters based on signeR results. Source data are provided in S20 Table. (PDF) [file pcbi.1009119.s016.pdf]

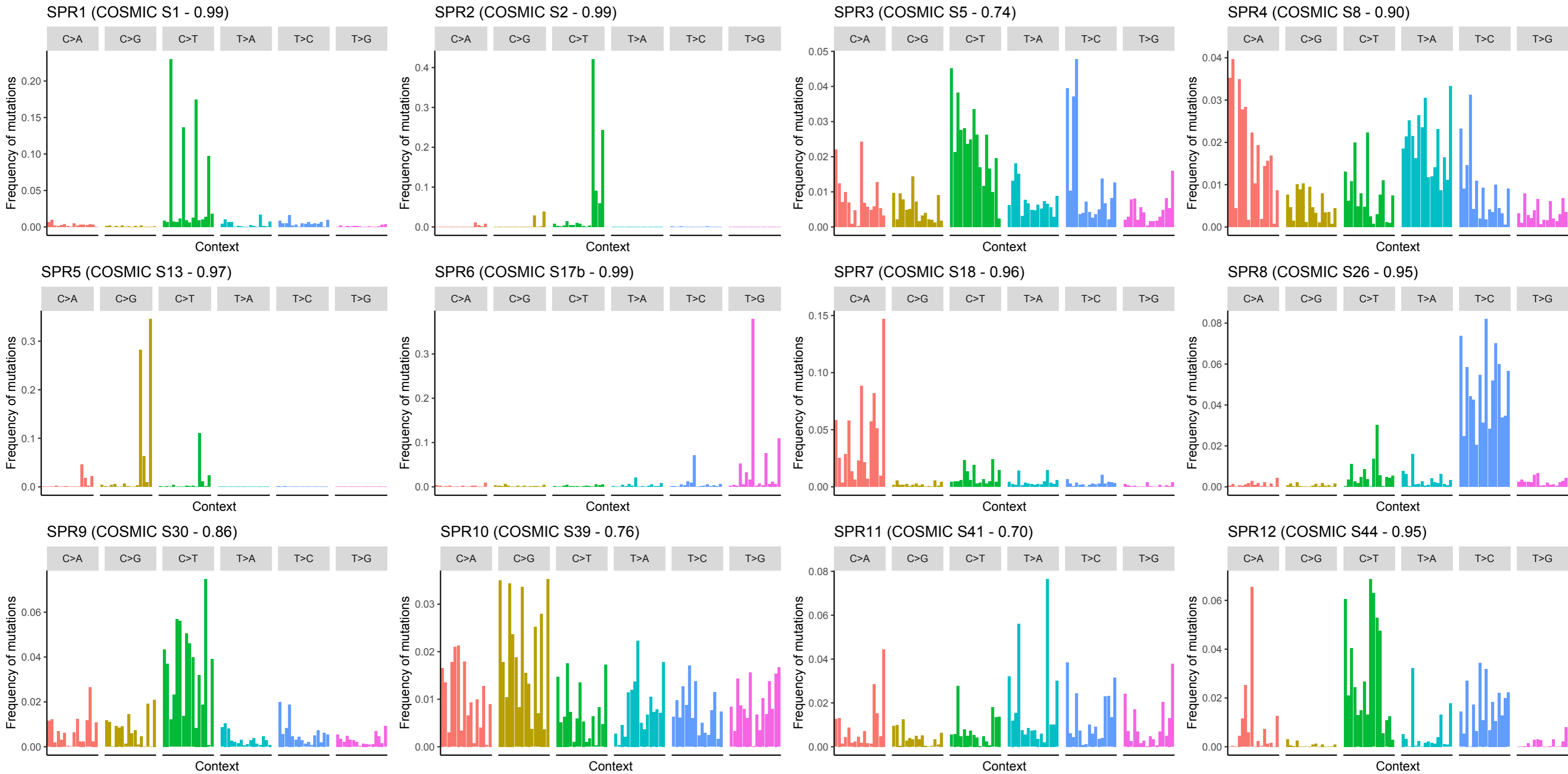

Supplement: S16 Fig — Source data are provided in S25 Table. (PDF) [file pcbi.1009119.s017.pdf]

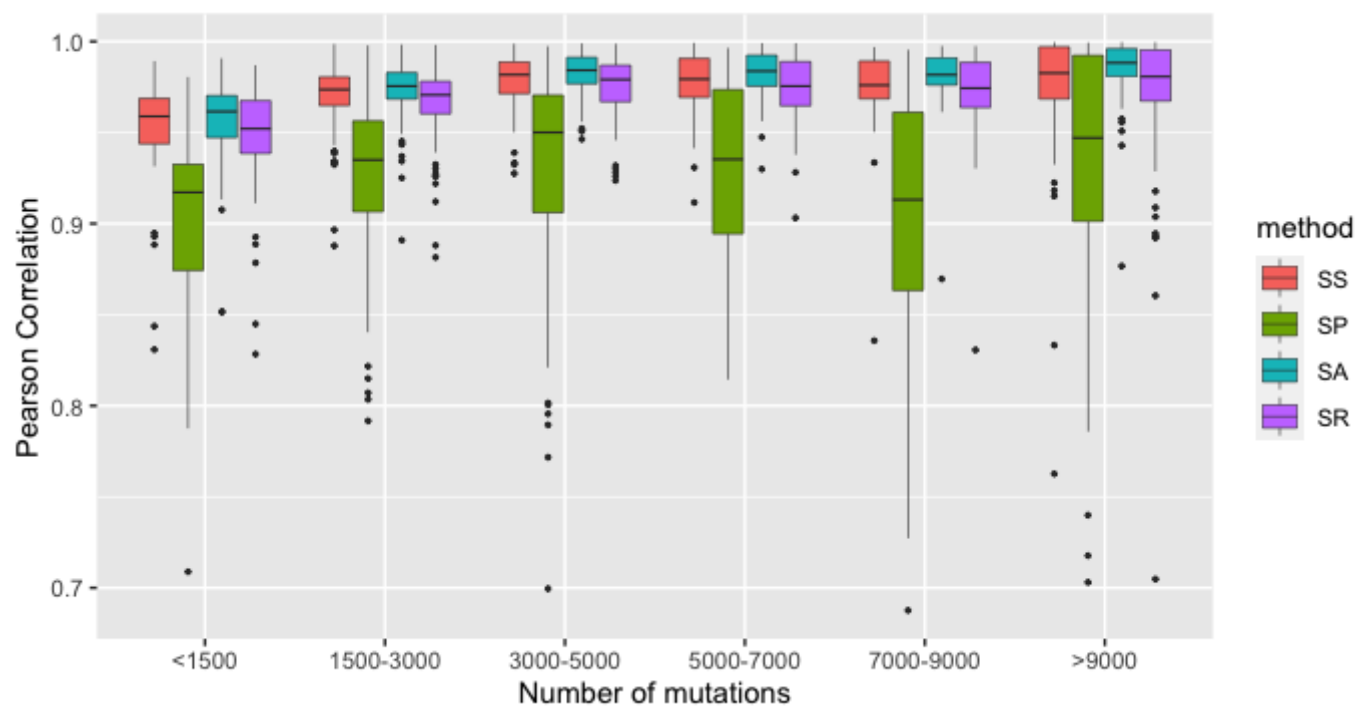

Supplement: S17 Fig — The x-axis shows the total number of mutations in the tumor. SS: SparseSignatures. SP: SigProfiler. SA: SignatureAnalyzer. SR: signeR. Source data are provided in S26 Table. (PDF) [file pcbi.1009119.s018.pdf]

SIR1 (COSMIC S1 - 0.86)

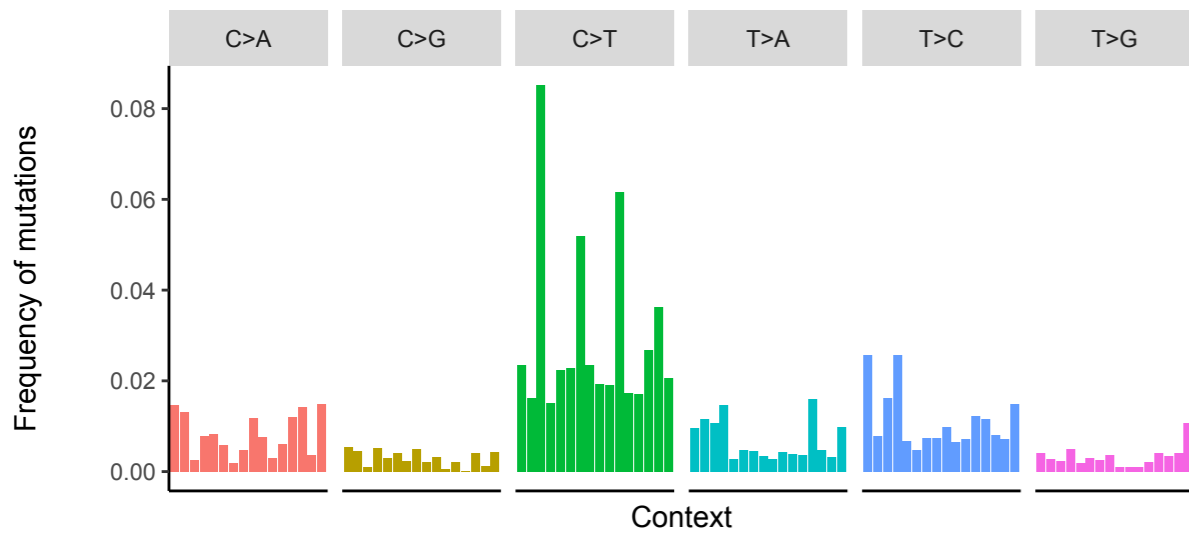

SIR2 (COSMIC S2 - 0.99)

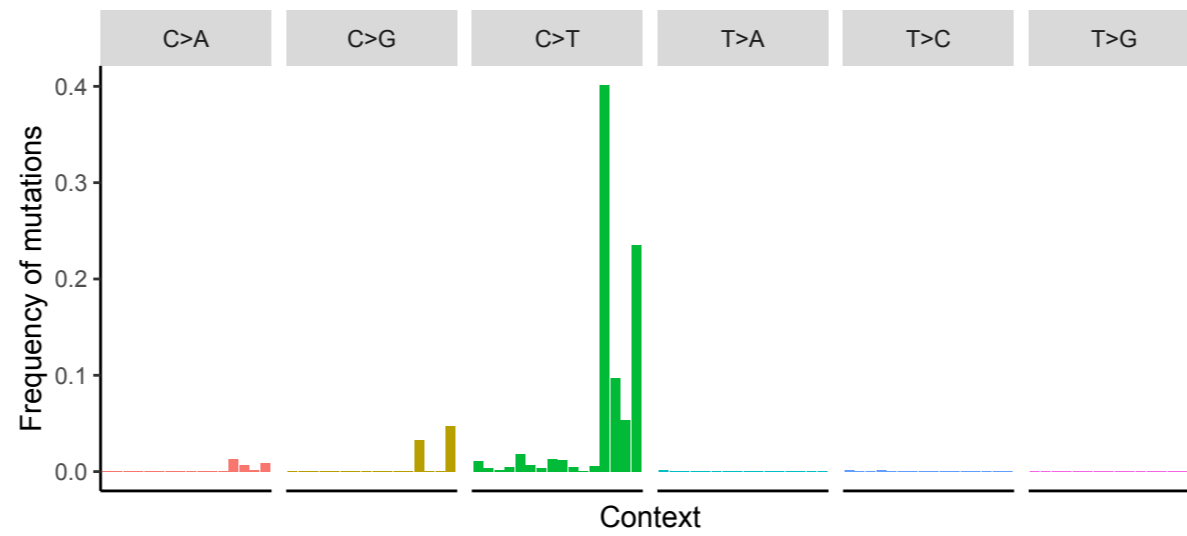

SIR3 (COSMIC S3 - 0.83)

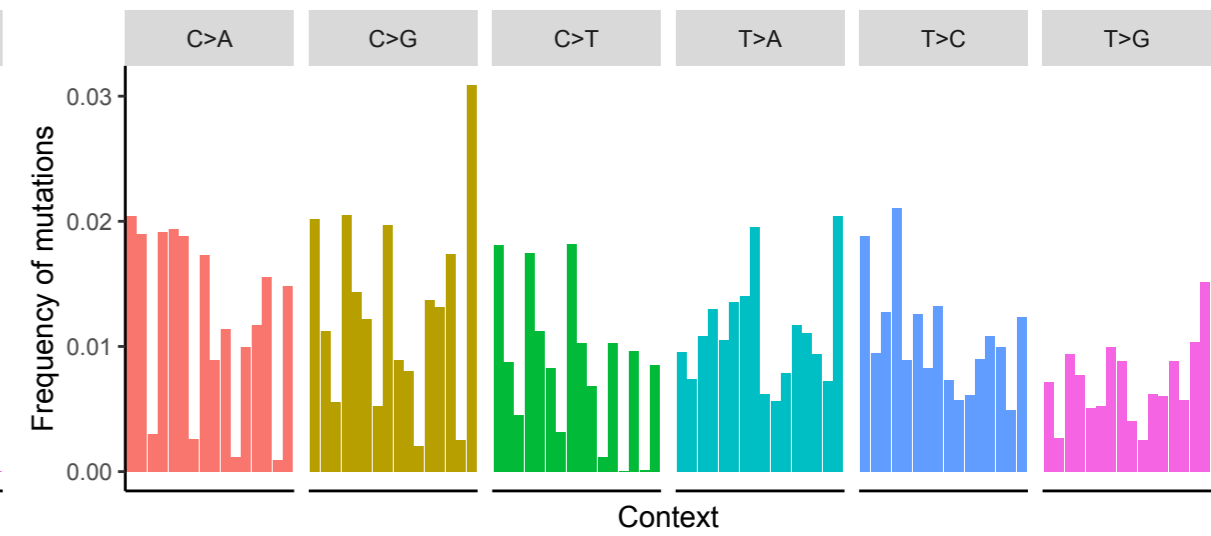

SIR4 (COSMIC S13 - 0.91)

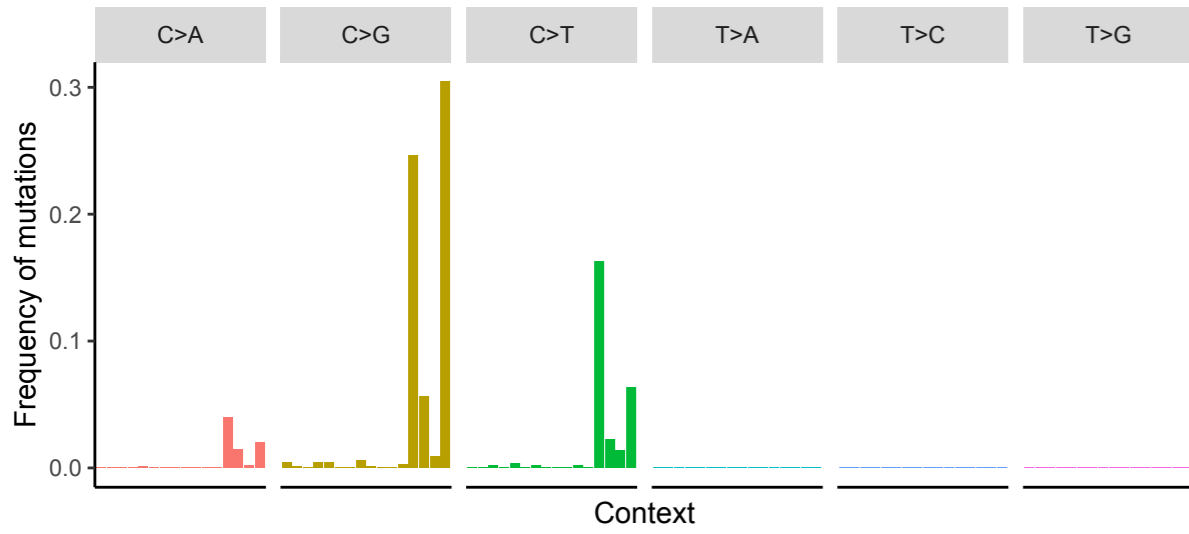

SIR5 (COSMIC S17b - 0.98)

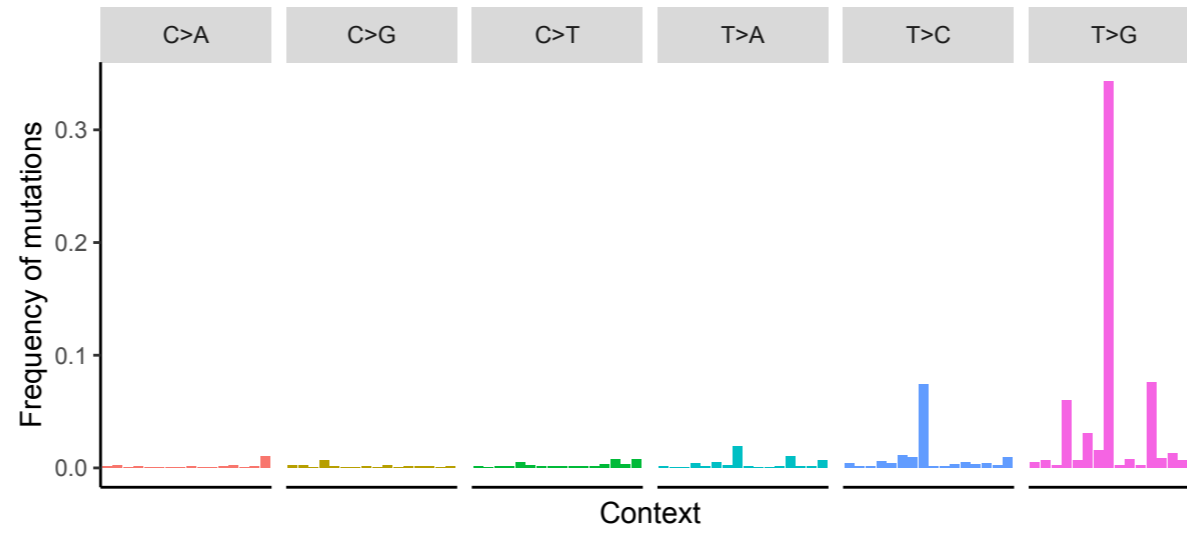

SIR6 (COSMIC S18 - 0.92)

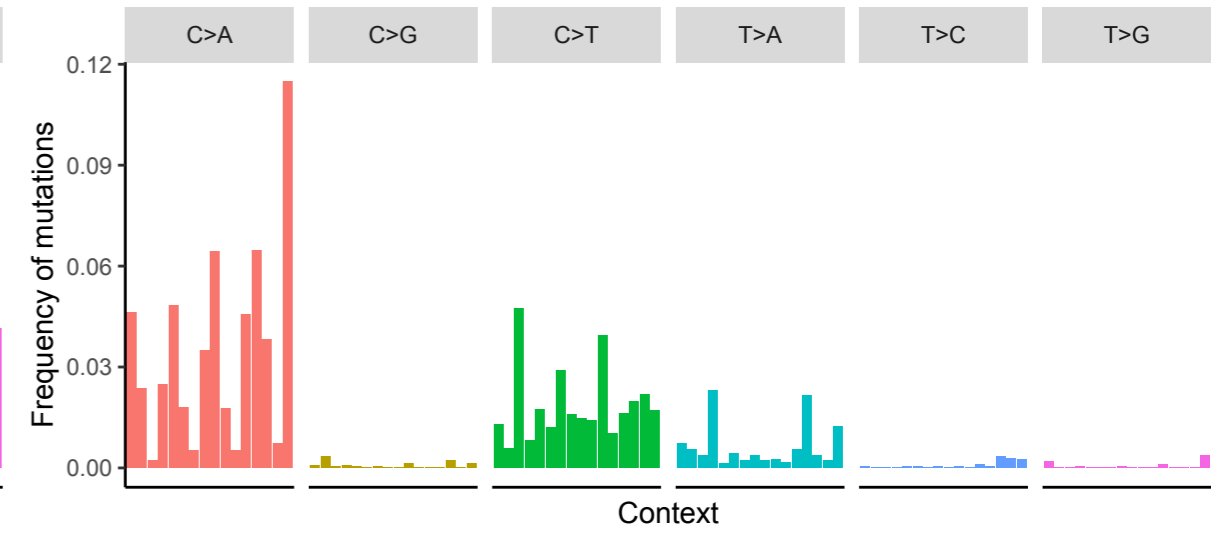

SIR7 (COSMIC S26 - 0.77)

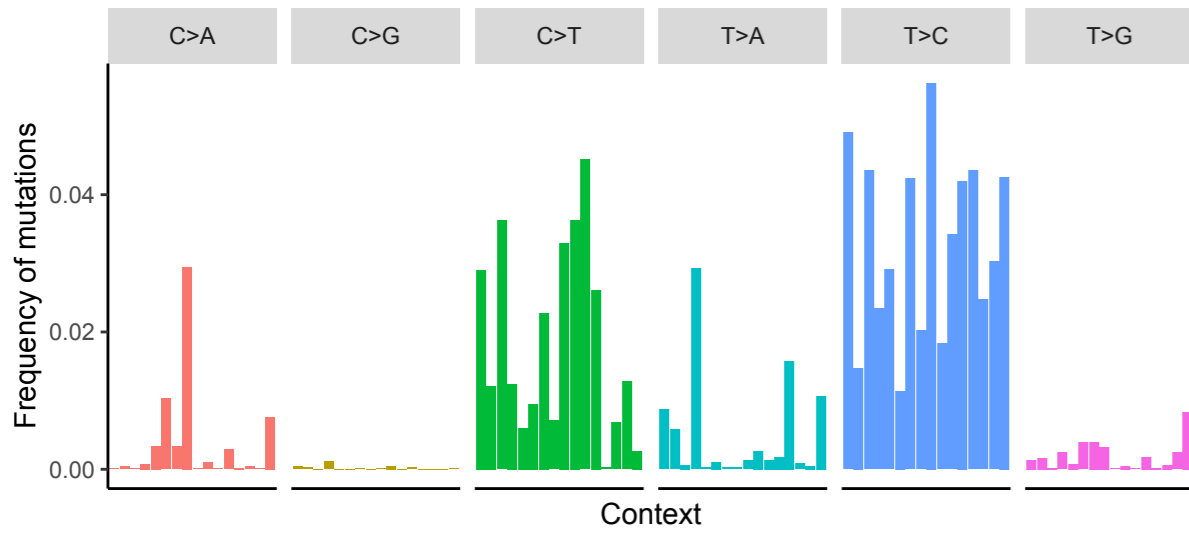

Supplement: S19 Fig — Source data are provided in S28 Table. (PDF) [file pcbi.1009119.s020.pdf]
