# Supplementary material for: De novo mutational signature discovery in tumor genomes using SparseSignatures
Source: PLoS Comput Biol. 2021 Jun 28;17(6):e1009119. doi: 10.1371/journal.pcbi.1009119 (PMC8270462; doi:10.1371/journal.pcbi.1009119)
Supplement: S8 Fig — A) Bar and line plot showing, for each method, the number of simulations in which it selected each value of K (number of signatures). The x-axis shows values of K and the y-axis shows the number of times each value was selected. Each method was run on 50 simulated datasets. In all cases, the correct value of K was 8. B) Box plots showing the residual error for the solutions produced by each method, over 50 simulations. Residual error was measured as the mean squared error (MSE) in reconstructing the original count matrix. C) Box plots showing the fraction of variance in the count matrix explained by the solutions produced by each method, over 50 simulations. D) Box plots showing the cosine similarity of reconstructing the 7 non-background input signatures, over 50 simulations. E) Box plots showing the mean squared error in reconstructing the exposure values for the 7 non-background input signatures, over 50 simulations. F) Box plots showing the sparsity of the signatures produced by each method, over 50 simulations. Sparsity was measured as the fraction of cells in the signature matrix whose value is <10−3. SS: SparseSignatures. SP: SigProfiler. SA: SignatureAnalyzer. SR: signeR. Source data are provided in S9 Table. (PDF) [file pcbi.1009119.s009.pdf]

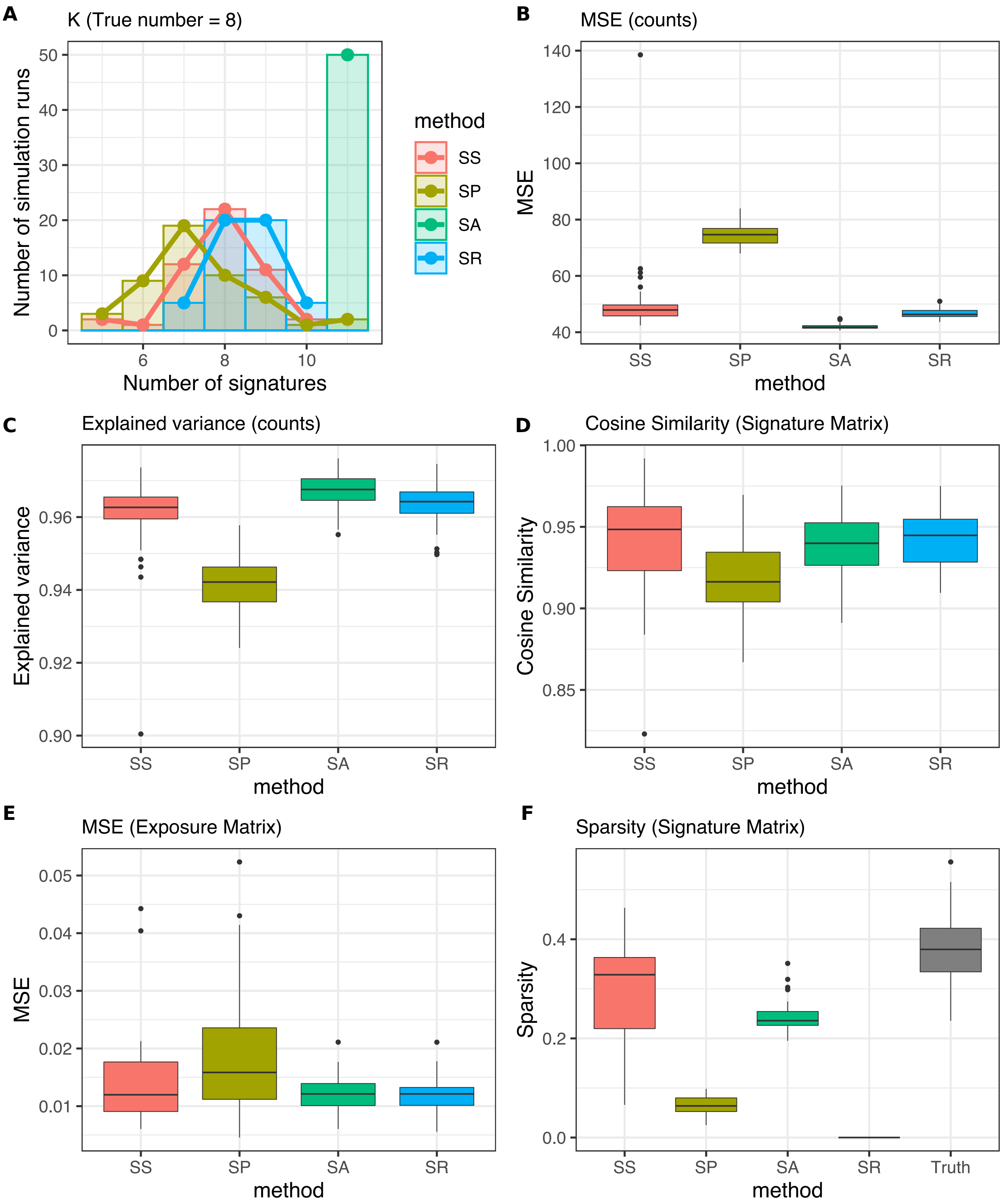
A
K (True number = 8)
Number of simulation runs
0
10
20
30
40
50
6
8
10
Number of signatures
method
SS
SP
SA
SR
B
MSE (counts)
MSE
40
60
80
100
120
140
SS
SP
SA
SR
method
C
Explained variance (counts)
Explained variance
0.90
0.92
0.94
0.96
SS
SP
SA
SR
method
D
Cosine Similarity (Signature Matrix)
Cosine Similarity
0.85
0.90
0.95
1.00
SS
SP
SA
SR
method
E
MSE (Exposure Matrix)
MSE
0.01
0.02
0.03
0.04
0.05
SS
SP
SA
SR
method
F
Sparsity (Signature Matrix)
Sparsity
0.0
0.2
0.4
SS
SP
SA
SR
Truth
method
