# Supplementary material for: De novo mutational signature discovery in tumor genomes using SparseSignatures
Source: PLoS Comput Biol. 2021 Jun 28;17(6):e1009119. doi: 10.1371/journal.pcbi.1009119 (PMC8270462; doi:10.1371/journal.pcbi.1009119)
Supplement: S18 Fig — Source data are provided in S27 Table. (PDF) [file pcbi.1009119.s019.pdf]

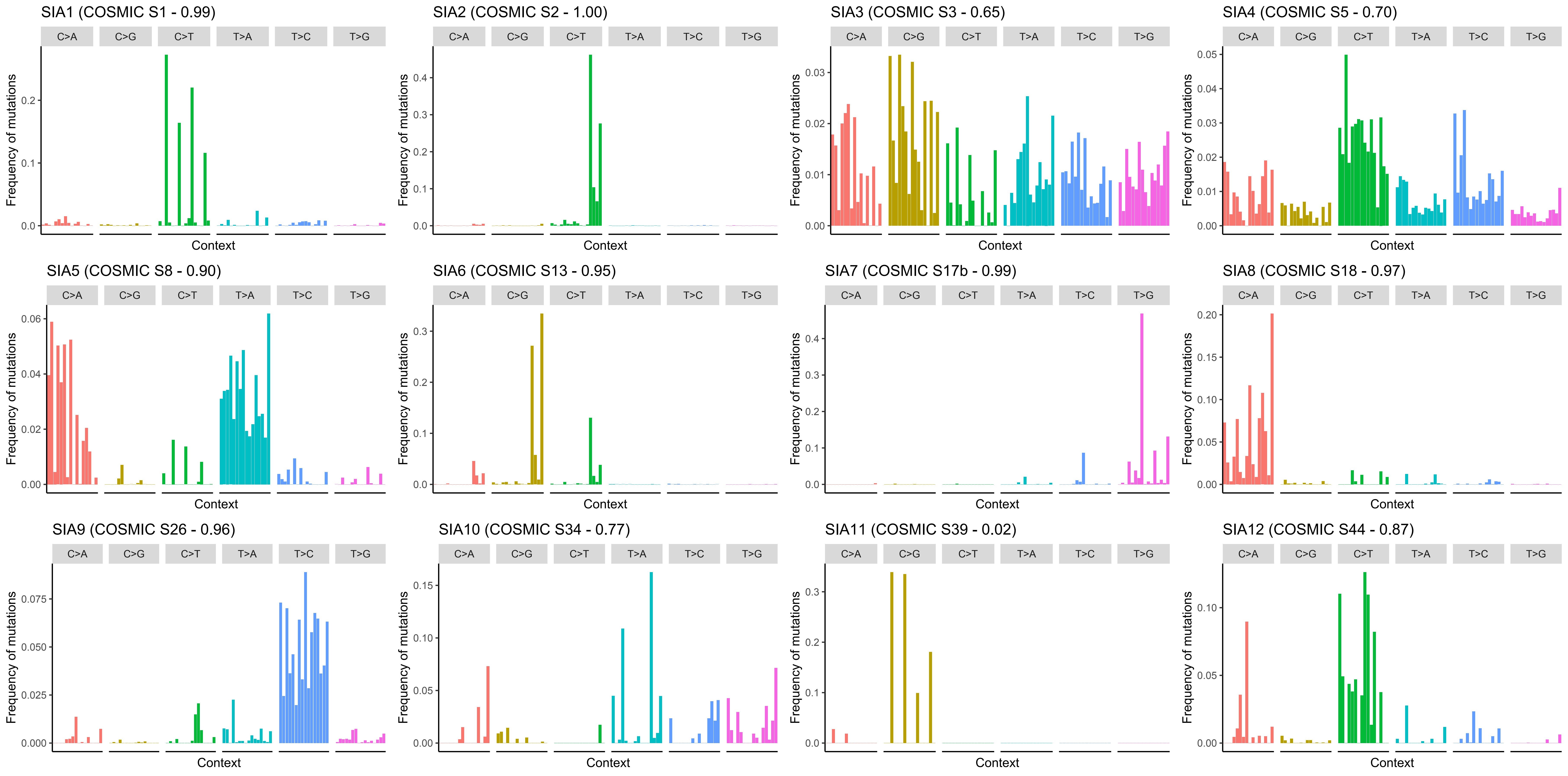

SIA1 (COSMIC S1 - 0.99)
SIA2 (COSMIC S2 - 1.00)
SIA3 (COSMIC S3 - 0.65)
SIA4 (COSMIC S5 - 0.70)
SIA5 (COSMIC S8 - 0.90)
SIA6 (COSMIC S13 - 0.95)
SIA7 (COSMIC S17b - 0.99)
SIA8 (COSMIC S18 - 0.97)
SIA9 (COSMIC S26 - 0.96)
SIA10 (COSMIC S34 - 0.77)
SIA11 (COSMIC S39 - 0.02)
SIA12 (COSMIC S44 - 0.87)
C>A
C>G
C>T
T>A
T>C
T>G
Frequency of mutations
Context
